# Pervasive homeobox gene function in the male-specific nervous system of *Caenorhabditis elegans*

**DOI:** 10.1242/dev.204958

**Published:** 2025-08-21

**Authors:** Robert W. Fernandez, Angelo J. Digirolamo, Giulio Valperga, G. Robert Aguilar, Laura Molina-García, Rinn M. Kersh, Chen Wang, Karinna Pe, Yasmin H. Ramadan, Curtis Loer, Arantza Barrios, Oliver Hobert

**Affiliations:** 1 Department of Biological Sciences, Columbia University, Howard Hughes Medical Institute, 1212 Amsterdam Avenue, New York, NY 10025, USA.; 2 Department of Cell and Developmental Biology, University College London, London WC1E 6BT, UK.; 3 University of San Diego, Department of Biology, San Diego, CA 92110, USA.

**Keywords:** *C. elegans*, Neuronal differentiation, Sexual differentiation, Homeobox gene

## Abstract

We explore here how neuronal cell type diversity is genetically delineated in the context of the large, but poorly studied, male-specific nervous system of the nematode *Caenorhabditis elegans.* Mostly during postembryonic development, the *C. elegans* male adds 93 male-specific neurons, falling into 25 cardinal classes, to the predominantly embryonically generated, sex-shared nervous system, comprising 294 neurons (116 cardinal classes). Using engineered reporter alleles, we investigate here the expression pattern of 40 of the 80 phylogenetically conserved *C. elegans* homeodomain proteins within the male-specific nervous system. Our analysis indicates that each individual neuron class is defined by unique combinations of homeodomain proteins and that the male-specific nervous system can be subdivided along the anterior/posterior axis in HOX cluster expression domains. Using a collection of newly available terminal fate markers, we undertake a mutant analysis of five homeobox genes (*unc-30*/Pitx, *unc-42*/Prop, *lim-6*/Lmx, *lin-11*/Lhx, *ttx-1*/Otx) and identify defects in cell fate specification and/or male copulatory defects in each of these mutant strains. Our analysis expands our understanding of the importance of homeobox genes in nervous system development and function.

## INTRODUCTION

The generation of molecular maps of animal brains has advanced tremendously over the past few years, with whole brain atlases now existing for several bilaterian model system species (e.g. Li et al., 2022; Taylor et al., 2021; Yao et al., 2023). Molecular brain maps raise a host of questions. For example, can the multidimensional (i.e. anatomical, molecular, functional) complexity of individual neuron types be reduced to a simple set of molecular descriptors? Are there common themes in the mechanisms that generate the enormous diversity of cell types that define each animal nervous system? A tentative answer to both of these questions has emerged in the brain of the nematode *Caenorhabditis elegans*. First, the analysis of expression of the entire family of homeodomain transcription factors (encoded by a total of 102 homeobox genes) has shown that each of the 118 distinct neuron classes of the nervous system of the hermaphrodite can be described by unique combinatorial codes of homeodomain expression (Reilly et al., 2020, 2022). Second, mutant analyses of homeobox genes over the past few decades have revealed that homeobox genes regulate the acquisition of specific neuronal identities – not just in *C. elegans*, but in many other animal species as well (reviewed by Hobert, 2021).

To investigate further how extensively homeobox genes define distinct neuronal cell types, we turned to the little explored nervous system of the *C. elegans* male. Based on sex-specific patterns of blast cell proliferation, sex-specific execution of cell death programs and sex-specific transdifferentiation, male animals generate an additional set of 93 neurons compared to the hermaphrodite, which are largely involved in sex-specific behaviors, such as copulatory behaviors (Cook et al., 2019; Emmons, 2014; Jarrell et al., 2012; Molina-Garcia et al., 2020; Sammut et al., 2015; Sulston et al., 1980). On the basis of lineage history, overall morphology and synaptic connectivity, these 93 neurons can be subdivided into 25 ‘cardinal classes’ (Cook et al., 2019; Emmons, 2014; Molina-Garcia et al., 2020; Sammut et al., 2015; Sulston et al., 1980) (Table 1). Two cardinal classes are located in the head (CEM and MCM neuron classes), two are located in the ventral nerve cord (CA and CP), and all others are located in several distinct ganglia in the tail of the animal, where they form a closely intertwined set of circuits that control various aspects of the complex male copulatory behavior (Emmons, 2018; Garcia and Portman, 2016; Liu and Sternberg, 1995). While many of the 25 cardinal classes are composed only of either unilateral neurons or bilateral neuron pairs, four cardinal classes – the CA and CP ventral cord neurons, and the tail ray sensory classes RnA and RnB – are subdividable into a multitude of different subclasses that are clearly distinguishable by connectivity and molecular markers (Lints and Emmons, 2002; Lints et al., 2004; Wang et al., 2024; Jarrell et al., 2012; Cook et al., 2019).

In contrast to the mostly embryonically generated, sex-shared nervous system of *C. elegans*, where neurons are generated after a series of rapid cell divisions, most male-specific neurons are generated from quiescent neuroblasts, generated initially as specialized epithelial cells in the embryo. In early larval stages, these neuroblasts lose their specialized epithelial features, re-enter the cell cycle and generate the vast majority of male-specific neurons, as well as other male-specific cell types (Sulston et al., 1980, 1983). These distinctive patterning mechanisms could trigger terminal differentiation programs that are distinct from those that generate sex-shared neurons during embryogenesis. In the most extreme version of such a scenario, these differentiation programs may rely less on the homeobox gene family that is so important in patterning the embryonic, sex-shared nervous system.

Only some very limited hints towards the involvement of homeobox genes in male-specific neurons exist. Male-specific CEM neurons were previously described to require the *unc-86/Brn3* POU homeobox gene for their proper differentiation (Nehme et al., 2010; Peden et al., 2007; Pereira et al., 2015; Shaham and Bargmann, 2002). In the ventral cord, the HOX cluster genes *lin-39/Scr* and *mab-5/Antp* control the differentiation of two classes of male-specific motor neurons and interneurons, the CA and the CP neurons, while the HOX cluster gene *egl-5/AbdB* controls the proper differentiation of a subset of ray sensory neurons (Clark et al., 1993; Kalis et al., 2014, 2022; Lints and Emmons, 1999; Salser et al., 1993). Beyond these hints of homeobox gene function in certain male-specific neurons, very little is known about the expression or function of homeobox genes in the many remaining male-specific neurons. We have set out here to address this gap in knowledge by making use of a large toolbox of strains that harbor *gfp* tags in their homeobox gene loci, a resource that we previously used to investigate homeodomain protein expression throughout the entire nervous system of the *C. elegans* hermaphrodite (Reilly et al., 2020, 2022).

Cellular sites of gene expression patterns in the male-specific nervous system have been notoriously difficult to identify based on the occasionally variable position of neuronal cell bodies in the male tail and the absence of reporter landmarks. This problem was recently overcome by the introduction of NeuroPAL, a multicolor fluorescent transgene in which each individual male-specific neuron class can be reliably identified through fluorescent landmarks in both the hermaphrodite and the male nervous system (Tekieli et al., 2021; Yemini et al., 2021).

Another problem, the paucity of well-described fluorescent reporter-based marker genes, has hampered the study of neuronal differentiation programs for which such markers are often of crucial importance. The recent systematic mapping of neurotransmitter identities in the male-specific nervous system has begun to mitigate this problem (Wang et al., 2024). In addition, our recent reporter-based analysis of neuropeptide-encoding genes in the hermaphrodite nervous system (Ripoll-Sanchez et al., 2023) has provided additional tools, in the form of neuropeptide reporter alleles, to identity more cell fate markers in the male-specific nervous system. We exploit these tools here to generate a collection of cell fate markers of male-specific neuron identities and to assess the impact of homeobox gene function on the differentiation programs of several male-specific neuron types. The analysis conducted here leads us to conclude that, despite the distinctive patterning mechanisms of male-specific neurons, homeobox genes play a role in male-specific neuron differentiation that appears to be as predominant as in the sex-shared nervous system.

## RESULTS

### Homeodomain protein expression analysis

The *C. elegans* genome codes for 102 homeobox genes, 80 of them conserved throughout the animal kingdom (Table S1). Since only an incomplete single-cell RNA sequencing (scRNA-seq) dataset exists so far for the male-specific nervous system of *C. elegans* (Haque et al., 2025; Morillo et al., 2025), we examined homeobox gene expression using a reporter gene approach, making use of a resource of *gfp*-tagged homeobox gene loci, expression of which we previously analyzed in the context of the hermaphrodite (Reilly et al., 2020, 2022). Apart from more complete coverage, this approach has the advantage over scRNA-seq analysis that the direct fusion of *gfp* to the respective homeobox gene visualizes protein, rather than mRNA expression, thereby capturing potential post-transcriptional gene regulatory events.

**Fig. 1.**
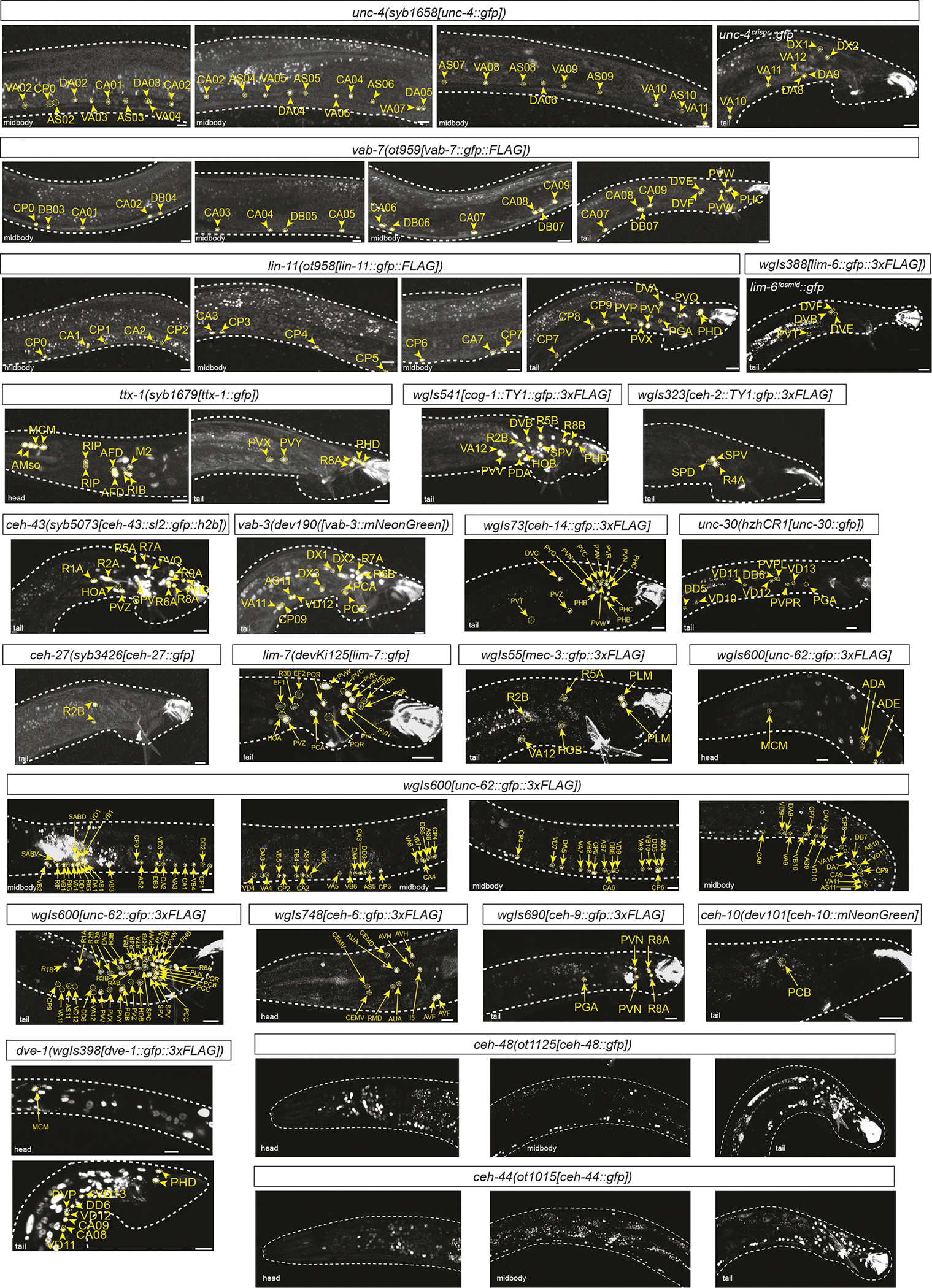
Representative images of homeobox reporter alleles. Representative images of homeobox reporters examined in this paper, which are either fosmid-based or CRISPR/Cas9-engineered alleles. Neuronal sites of expression were identified through overlap with NeuroPAL signals (Tekieli et al., 2021; Yemini et al., 2021). White dashed lines are used to trace the contours of the animal. The corresponding images showing overlap with NeuroPAL colors are in Fig. S1, with the exception of the pan-neuronal *ceh-44* and *ceh-48*. Expression patterns are summarized in Table 1. Images are representative of 4–10 samples. Scale bars: 10 μm.

We analyzed the expression of half (40) of the 80 conserved homeodomain proteins, covering all main homeodomain subclasses (Antp-like, Prd-like, POU, LIM, SIX, etc.). We assessed expression of all male-specific neurons in the head, ventral nerve cord and tail (Fig. 1, Table 1, Table S1). For identification of the neuronal sites of expression, we used the NeuroPAL transgene, which differentially labels all neuron classes in the male-specific nervous system (Tekieli et al., 2021) (Fig. S1).

For 12 of the examined 40 homeodomain proteins, we observed no expression in male-specific neurons (summarized in Table S1). As expected from their pan-neuronal expression in the hermaphrodite (Leyva-Diaz and Hobert, 2022), two genes, the CUT homeobox genes *ceh-44* and *ceh-48*, were expressed throughout the entire male-specific nervous system (Fig. 1). All other 26 homeodomain proteins showed restricted expression in subsets of male-specific neurons (Fig. 1, Table 1). We found that each male-specific neuron class expressed at least one (non-pan-neuronal) homeodomain protein, therefore matching the complete coverage of all neuron classes in the hermaphrodite by homeobox genes (Reilly et al., 2020). Most homeobox genes were expressed in small subsets of male-specific neuron classes, sometimes exclusively in single-neuron classes (*unc-42/Prop1* in MCM, *unc-30/Pitx* in PGA). Exceptions to very restricted homeodomain proteins patterns were the six HOX cluster proteins, as well as their frequent co-factor UNC-62/Meis, each of which were broadly, yet still highly cell type-specifically, expressed throughout the male-specific nervous system. Other than two of the AbdB-type HOX cluster genes, *php-3* and *nob-1*, which recently duplicated specifically in the *Caenorhabditis* genus (Kirangwa et al., 2024), no two homeobox genes showed the exact same expression pattern (Fig. 1, Table 1).

Each male-specific neuron co-expressed, on average, three homeobox genes from the analyzed dataset (range: one to seven, excluding the pan-neuronal CUT homeobox genes). There appeared to be no preferential partnership of any two homeodomain proteins, with the exception of the frequent association of UNC-62/Meis expression with a HOX cluster gene, expected from the evolutionarily ancient biochemical partnership of HOX and Meis proteins (Merabet and Galliot, 2015). However, there were also clear cases in which either HOX or Meis proteins were expressed independently of one another, as has also been observed in other organisms (Moens and Selleri, 2006). The homeobox gene combinations that we observed in the male tail were notably distinct from those in other parts of the nervous system (Reilly et al., 2020), largely owing to the prominence of HOX cluster gene expression in the male-specific nervous system, compared to the sex-shared anterior nervous system. We describe HOX protein expression patterns in more detail in a separate section below.

Strikingly, of the cardinal 25 male-specific neuron classes, there are only two sets of neuron classes that cannot presently be distinguished by unique combinations of the homeobox genes (even though they can be distinguished by neuropeptides; Table 2): The lineally related PCA and PCC share the same homeobox code (*ceh-43* and *vab-3*) as do the lineally related PVX and PVY (*lin-11* and *ttx-1*, plus three HOX cluster genes) (Table 1). As reflected by their shared name (and their shared lineage), the PCA and PCC neuron classes, as well as the PVX and PVY neuron classes are also anatomically similar to one another (Cook et al., 2019; Jarrell et al., 2012). Nevertheless, since we only analyzed half of all conserved *C. elegans* homeobox genes, we anticipate that a complete examination may attach unique homeodomain codes to these neuron classes.

Within each cardinal class that can be clearly subdivided into subtypes (RnA, RnB, CAn and CPn neurons), we also identified homeobox codes for several, but not all, molecularly previously defined subtypes (Fig. 1, Table 1). Specifically, within the ventral nerve cord, we observed that homeobox genes subdivide male-specific CA and CP neuron classes into subclasses, in accordance with earlier studies using cell fate markers (Kalis et al., 2014) and recent neurotransmitter mapping studies (Wang et al., 2024). Similarly, in the male tail, the A- and B-type ray neuron classes, each composed of nine class members, could be subdivided into various subclasses based on homeobox gene expression (Fig. 1, Table 1), again in accordance with earlier studies using other markers (Lints et al., 2004; Wang et al., 2024).

We discovered previously unidentified subtypes within the DX and EF neuron classes, each of which is composed of up to four class members (*lim-7/Islet*: EF1/2, not EF3/4; *unc-4:* DX1/2, not DX3/4). Conversely, cardinal neuron classes with previously much appreciated subclass diversity are ‘unified’ by the expression of particular homeobox genes: all CA neurons expressed the Eve/Evx-type homeobox gene *vab-7*, all CP neurons expressed the *lin-11* LIM homeobox gene and all A-type ray neurons expressed the Dlx-type homeobox gene *ceh-43* (Fig. 1, Table 1).

### HOX cluster genes show anteriorly/posteriorly patterned expression in the male-specific nervous system

**Fig. 2.**
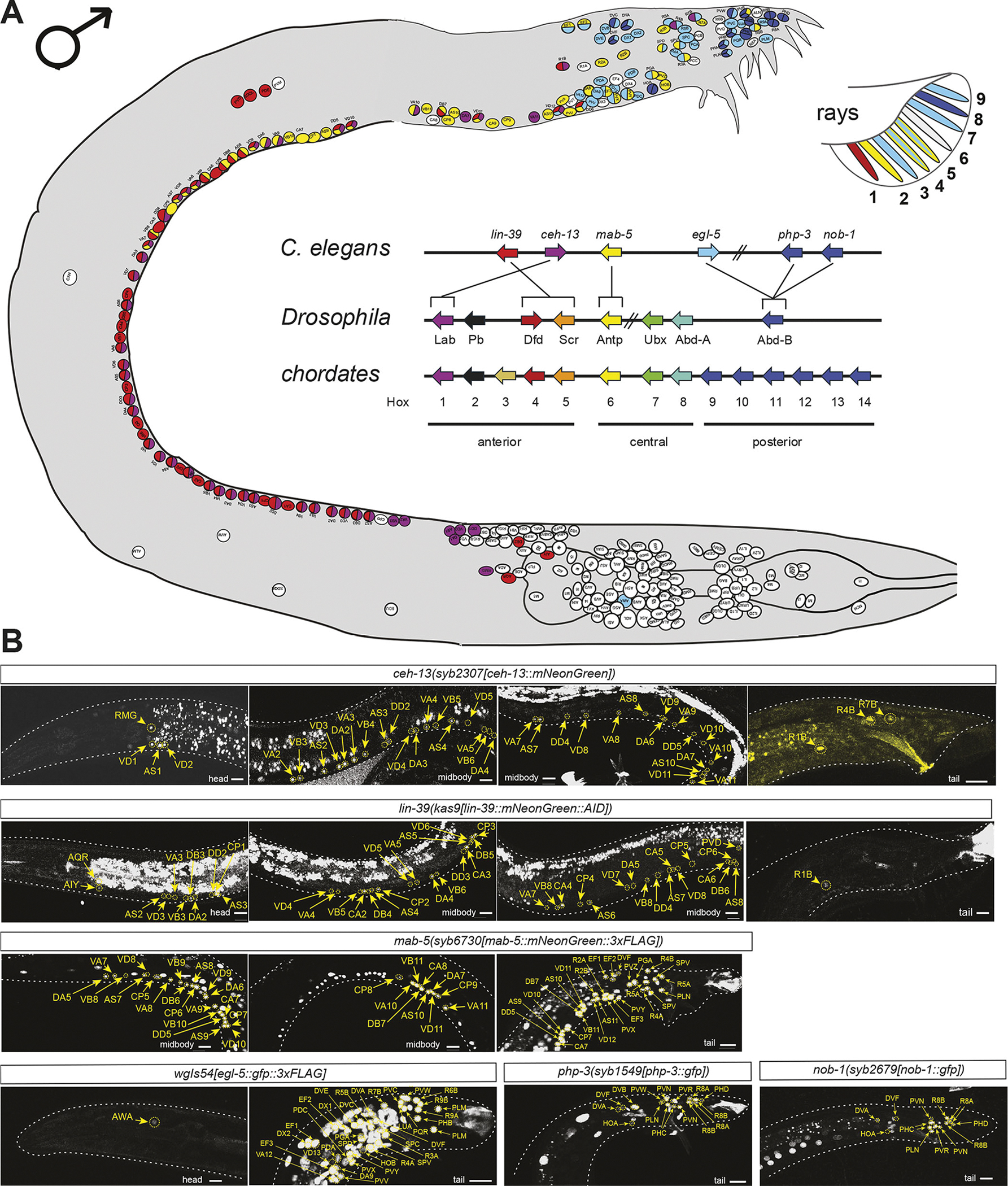
Expression of HOX cluster genes. (A) Diagram of the expression pattern of HOX genes in male *C. elegans*. The genomic positions of HOX genes from *C. elegans*, *Drosophila* and chordates are also represented. HOX cluster gene similarities are taken from Hueber et al. (2010). (B) Expression of CRISPR/Cas9 engineered reporter alleles and fosmid shows the spatially controlled expression of HOX proteins. Panels show representative images of each protein expression pattern. Images are representative of 4–10 samples. Circles indicate the HOX protein signal, and arrows are used to match the signal to the specific neural ID. Gut autofluorescence is visible in images for *ceh-13(syb2307)* and *lin-39(kas9)*. Autofluorescence from the male tail is visible in reporters for *lin-39(kas9)*, *mab-5(syb6730)*, *egl-5* (reporter array *wgIs54*) and *php-3(syb1549)*. White dashed lines are used to trace the contours of the animal. To obtain a definitive neural ID, multiple images acquired with the NeuroPAL landmark (*otIs669* or *otIs696*) were used to obtain a neural ID overlaid on representative images. The corresponding images showing overlap with NeuroPAL colors are in Fig. S2. Scale bars: 10 μm.

One set of homeobox genes that warrant a separate consideration are the *C. elegans* HOX cluster genes (Fig. 2). In the context of animal nervous systems, the analysis of HOX gene expression and function has largely focused on the ventral nerve cord of invertebrates and spinal cord of vertebrates, in which HOX cluster genes are differentially expressed along the anterior/posterior axis, showing a remarkable match to their chromosomal localization (‘co-linearity’; reviewed by Alexander et al., 2009; Krumlauf, 2018; Philippidou and Dasen, 2013; Smith and Kratsios, 2024). In the *C. elegans* hermaphrodite, HOX gene expression extends posteriorly beyond the ventral nerve cord into various tail ganglia, which express the AbdB-homologs *egl-5*, *php-3* and *nob-1* (Kratsios et al., 2017; Reilly et al., 2020; Smith et al., 2024). In the male, the expression of a subset of HOX cluster genes had already been examined in some restricted regions of the male tail (e.g. Ferreira et al., 1999; reviewed by Smith and Kratsios, 2024), but no comprehensive and comparative analysis was yet available. Using reporter alleles for all HOX cluster genes, we note the following themes of HOX gene expression in the adult male nervous system (Fig. 2B, Fig. S2, Table 1). (1) The vast majority of mature, male-specific neurons in the ventral cord and tail ganglia express at least one HOX cluster gene. This is in striking contrast to neurons in the anterior head ganglia, which express very few HOX cluster genes (Fig. 2B). (2) Along the male ventral nerve cord, the postembryonically added, male-specific CA and CP neurons show similar patterns as the sex-shared neurons: concordant with their chromosomal location, *lin-39* and *mab-5* show a spatially co-linear expression with *lin-39* being expressed in more anterior CA and CP class members, and *mab-5* in more posterior CA and CP class members. (3) In male tail ganglia, which contain a manifold increase in neuron number of all different types (sensory, inter- and motor neurons) compared to hermaphrodites, domains of HOX gene expression still show a notable co-linearity with their genomic arrangement: there is no expression of the anterior HOX cluster gene *lin-39*, while *mab-5* expression is observed in more anterior parts of these ganglia but then peters out. *egl-5* expression predominates, with the most posterior *AbdB* paralogs *nob-1* and *php-3* being more enriched in the most posterior neurons (Fig. 2B, Table 1). (4) Spatial co-linearity of HOX gene expression is recapitulated, albeit imperfectly, in the ‘microcosm’ of the ray neurons, which innervate the sensory ray structures of the worm that are aligned in an anterior-to-posterior manner along the male bursa (Fig. 2A, inset). (5) The *ceh-13/Lab* homeobox gene represents a curious case in the *C. elegans* HOX cluster. *ceh-13* is the *C. elegans* homolog of the most anterior HOX cluster gene of other metazoans, Labial in flies and *HoxA/B/C/D1* in vertebrates, but it has switched its chromosomal order with *lin-39*, the *C. elegans* Dfd/Scr homolog (Fig. 2A) (Bürglin and Ruvkun, 1993; Kirangwa et al., 2024). Nevertheless, in the embryo, the *ceh-13* expression pattern is indeed enriched in the anterior part (Brunschwig et al., 1999; Wittmann et al., 1997). However, unlike the other HOX cluster genes, an anterior/posterior gradient is not evident in the context of the hermaphrodite ventral nerve cord (Reilly et al., 2020). In males, *ceh-13* is expressed in several ray neurons in the tail.

### Temporal dynamics of homeodomain protein expression in the male-specific nervous system

**Fig. 3.**
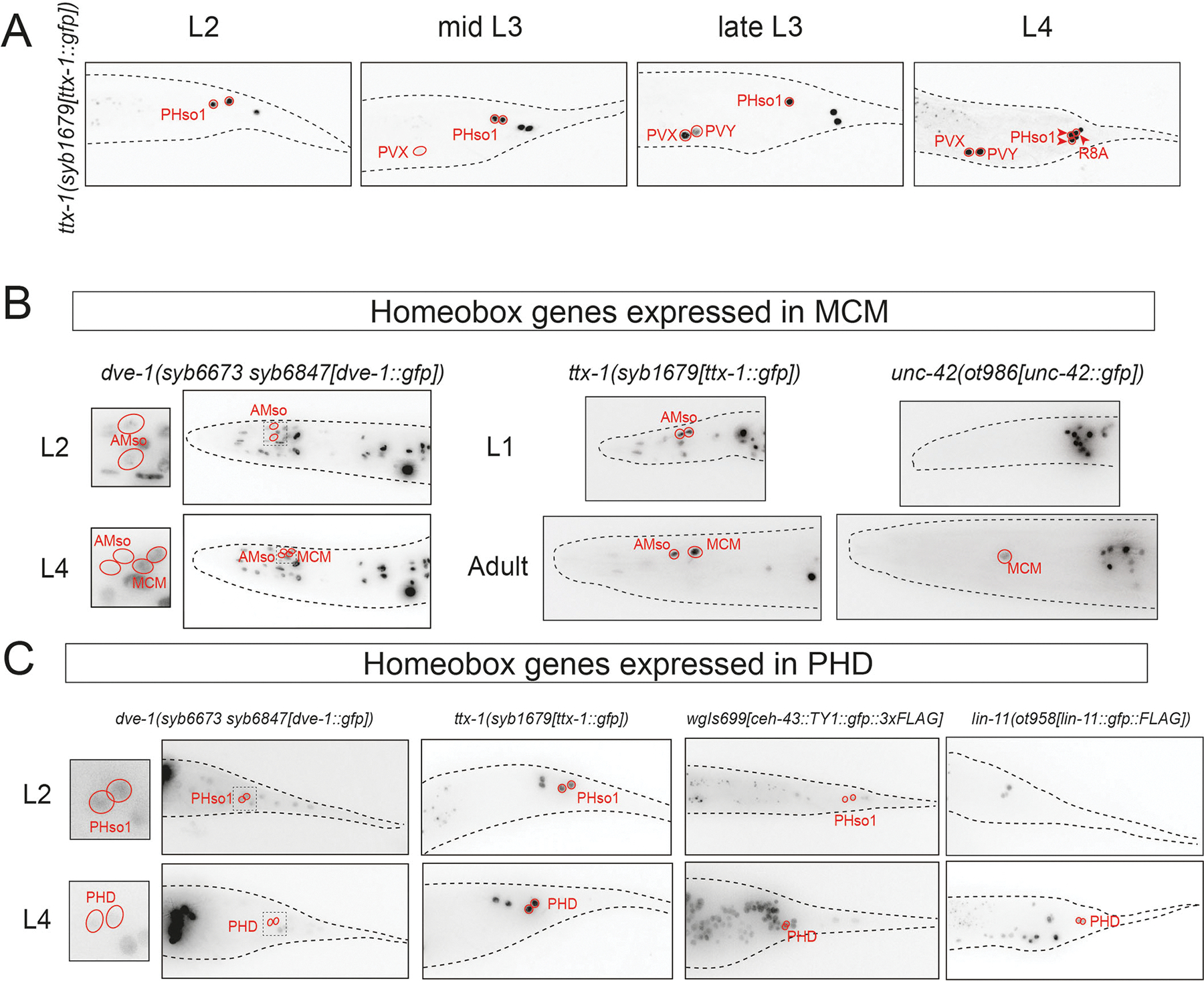
Temporal dynamics of homeobox gene expression in male neurons relative to neuronal transdifferentiation. (A) Temporal dynamics of *ttx-1(syb1679)* expression in the neurons of the male tail. Although PVX is generated in the first larval stage (Sulston et al., 1980), expression of TTX-1 in PVX was only detectable by the third larval stage. (B) Temporal dynamics of homeobox genes expressed in MCM. *dve-1(syb6673 syb6847)* and *ttx-1(syb1679)* are expressed in the glial precursors of MCM (AMso) and continue to be expressed in both AMso and MCM after the transdifferentiation event at sexual maturation. *unc-42(ot986)* was expressed only in MCM and not its glial precursor AMso. In B and C, the boxed region in the *dve-1* panels is magnified for clarity. (C) Temporal dynamics of homeobox genes expressed in PHD. *dve-1(syb6673 syb6847)*, *ttx-1(syb1679)* and the fosmid-based *ceh-43* reporter *wgIs699* were expressed in the glial precursors of PHD (PHso1) and continued to be expressed in PHD after the transdifferentiation event at sexual maturation. PHso1 to PHD transdifferentiation is direct and does not involve cell division. *lin-11(ot958)* was expressed only in PHD and not in its glial precursor PHso1. Images are representative of 8–10 samples. Black dashed lines are used to trace the contours of the animal.

Our expression analysis of all homeodomain proteins has focused on the fully mature nervous system. While we have not systematically examined the onset of expression of homeodomain proteins during developmental specification, we note the presence of widely divergent onsets of expression. Previous work has revealed expression of the AbdB-type EGL-5 HOX already in the epithelial neuroblast precursor of many male-specific neurons at the first larval stage (Ferreira et al., 1999), mirrored by neuroblast division defects observed in *egl-5* mutants (Chisholm, 1991). Contrasting this precursor expression, we did not observe *unc-30* expression in the P11p lineage during the L3 stage, during which the P11.p divides to generate several neurons, including the eventually *unc*-30-expressing PGA neuron. On the extreme end of the spectrum, we found that the TTX-1 homeodomain protein only becomes visible in the P12-derived PVX neuron by the third larval stage (Fig. 3A), even though this neuron is already generated after a few divisions of the P12 neuroblast in the first larval stage (Sulston et al., 1980).

We observed two distinct onsets of homeodomain expression in two male-specific neuron classes generated by transdifferentiation. The two male-specific neuron classes, the head interneuron MCM and tail sensory neuron PHD, are generated during sexual maturation through a transdifferentiation process from glial cell types, the AMso glia in the case of MCM and the PHso1 glia in the case of PHD (Molina-Garcia et al., 2020; Sammut et al., 2015). We found that some homeodomain proteins are already expressed in the socket glia from which these neurons are generated: MCM-expressed *dve-1* and *ttx-1* were expressed already in the AMso glia precursor (Fig. 3B), while PHD-expressed *ceh-43* was expressed already in PHso1 (Fig. 3C). Two other homeodomain proteins, *unc-42* and *lin-11*, were, however, only expressed in the respective neuron upon transdifferentiation from glia to neurons: *unc-42* was expressed in MCM, but not its AMso precursor (Fig. 3B), and *lin-11* was expressed in PHD, but not PHso1 (Fig. 3C).

### A toolbox of terminal differentiation markers for male-specific neurons

**Fig. 4.**
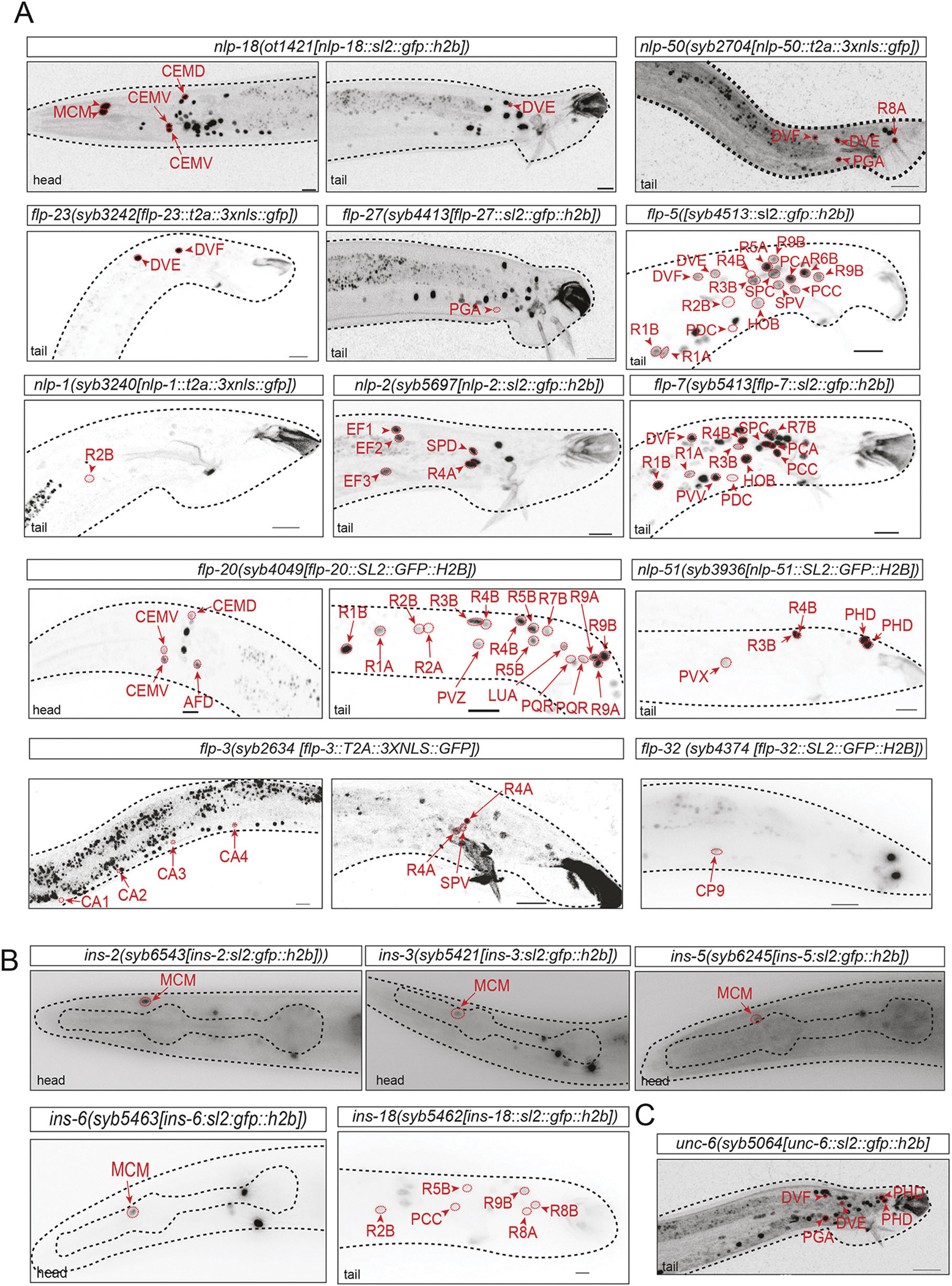
Terminal differentiation markers for male-specific neurons. (A) CRISPR/Cas9-engineered SL2::GFP::H2B-based reporter alleles reveal expression of several neuropeptides in male-specific neurons. Additional reporter allele data that is not shown here is summarized in Table 2. (B) Expression of CRISPR/Cas9-engineered SL2::GFP::H2B-based reporter alleles of insulin-like peptides in the male nervous system. Four of these, *ins-2(syb6543)*, *ins-3(syb5421)*, *ins-5(syb6245)* and *ins-6(syb5463)*, show expression in MCM but not in other neurons in the male nervous system. *ins-18(syb5462)* is expressed in several neuron types in the male tail. (C) The CRISPR/Cas9-engineered *unc-6(syb5064)* reporter allele is expressed in DVE, DVF, PHD and PGA neurons belonging to distinct ganglia (preanal, lumbar, dorsorectal). Images are representative of 3–9 samples. Black dashed lines are used to trace the contours of the animal and its pharynx. For all panels, the corresponding images showing overlap with NeuroPAL (*otIs669* or *otIs696*) colors are in Fig. S3. Scale bars: 10 μm.

One reason why so few previous studies have examined the regulation of differentiation programs in the male-specific nervous system has been the paucity of molecular markers for most male-specific neurons. The construction of the NeuroPAL transgene, which combines a multitude of differentiation markers for the male-specific nervous system (Tekieli et al., 2021), as well as our recent mapping of neurotransmitter identities of male-specific neurons (Wang et al., 2024), has improved the situation, but for many neuron classes still only a limited number of differentiation markers are available (Kalis et al., 2014; Lints et al., 2004). To generate more markers for an analysis of homeobox gene function in the male-specific nervous system, we analyzed the expression of neuropeptide-encoding genes, which display strong and highly neuron type-selective expression throughout the hermaphrodite nervous system, as revealed by previous reporter gene and scRNA-seq analysis (Kim and Li, 2004; Ripoll-Sanchez et al., 2023; Taylor et al., 2021). We scanned through a set of genome-engineered neuropeptide reporter alleles available in our lab and settled on a more precise investigation of 17 neuropeptide-encoding genes, including seven FLP, six NLP and five insulin-like peptides. We analyzed their expression in the male-specific nervous system using the NeuroPAL transgene and discovered highly cell type-specific patterns for each of them, covering in aggregate a large majority of the male-specific nervous system (Fig. 4, Fig. S3, Table 2). Of particular note is the selective co-expression of four insulin-like genes exclusively in the MCM neuron in the head of adult males (with no expression elsewhere in the male-specific nervous system) (Fig. 4B, Table 2).

In addition to these 17 neuropeptide-encoding genes, we also identified the male-specific sites of expression of *unc-6/Netrin*, neuron type-specific expression of which has served as a valuable cell fate marker for several sex-shared head neuron classes (Berghoff et al., 2021) (Fig. 4C, Table 2). We found that *unc-6* is selectively expressed in four male-specific neuron classes (DVE, DVF, PHD, PGA), covering three different tail ganglia ( preanal, lumbar, dorsorectal). Expression was observed not just transiently, as one would perhaps expect from a gene so prominently involved in axon pathfinding (Chisholm et al., 2016), but was rather stably observed as males reached maturity. Such late expression is suggestive of UNC-6/Netrin function in, for example, synaptic wiring.

### The Pitx homolog *unc-30* is required for PGA interneuron differentiation

Armed with these molecular markers, we set out to examine whether homeobox genes affect neuronal differentiation to an extent similar to what has been observed in other neuronal contexts in the hermaphrodite. We first examined one of the two most sparsely expressed homeobox genes, the *C. elegans* homolog of the vertebrate Pitx genes, *unc-30*, which, in the context of the male-specific nervous system, is exclusively expressed in a single male-specific neuron class, the PGA interneuron in the preanal ganglion. PGA is one of a total of four pre-anal ganglion interneurons generated by the P11 epidermal blast cells in response to Wnt and EGF signaling (Yu et al., 2009). PGA is one of the few neurons in the *C. elegans* nervous system that expresses multiple neurotransmitter systems: based on *unc-17/VAChT* expression, PGA is cholinergic but also uses an additional, unknown neurotransmitter, based on expression of the GABA/glycine vesicular transporter *unc-47/VGAT* and the concomitant absence of *unc-25/GAD* (Wang et al., 2024). Moreover, PGA uptakes and utilizes serotonin, as inferred from the absence of *tph-1/TPH* expression, 5-hydroxytryptamine (5-HT) antibody staining, *cat-1/VMAT* expression and *mod-5/SERT* expression (Wang et al., 2024). In addition, our marker analysis described above identified two neuropeptides (*flp-27* and *nlp-50*) expressed in PGA, as well as the axon guidance/synaptogenic *unc-6* gene (Fig. 4A,C). PGA is also marked by a specific color-code in NeuroPAL (Tekieli et al., 2021).

**Fig. 5.**
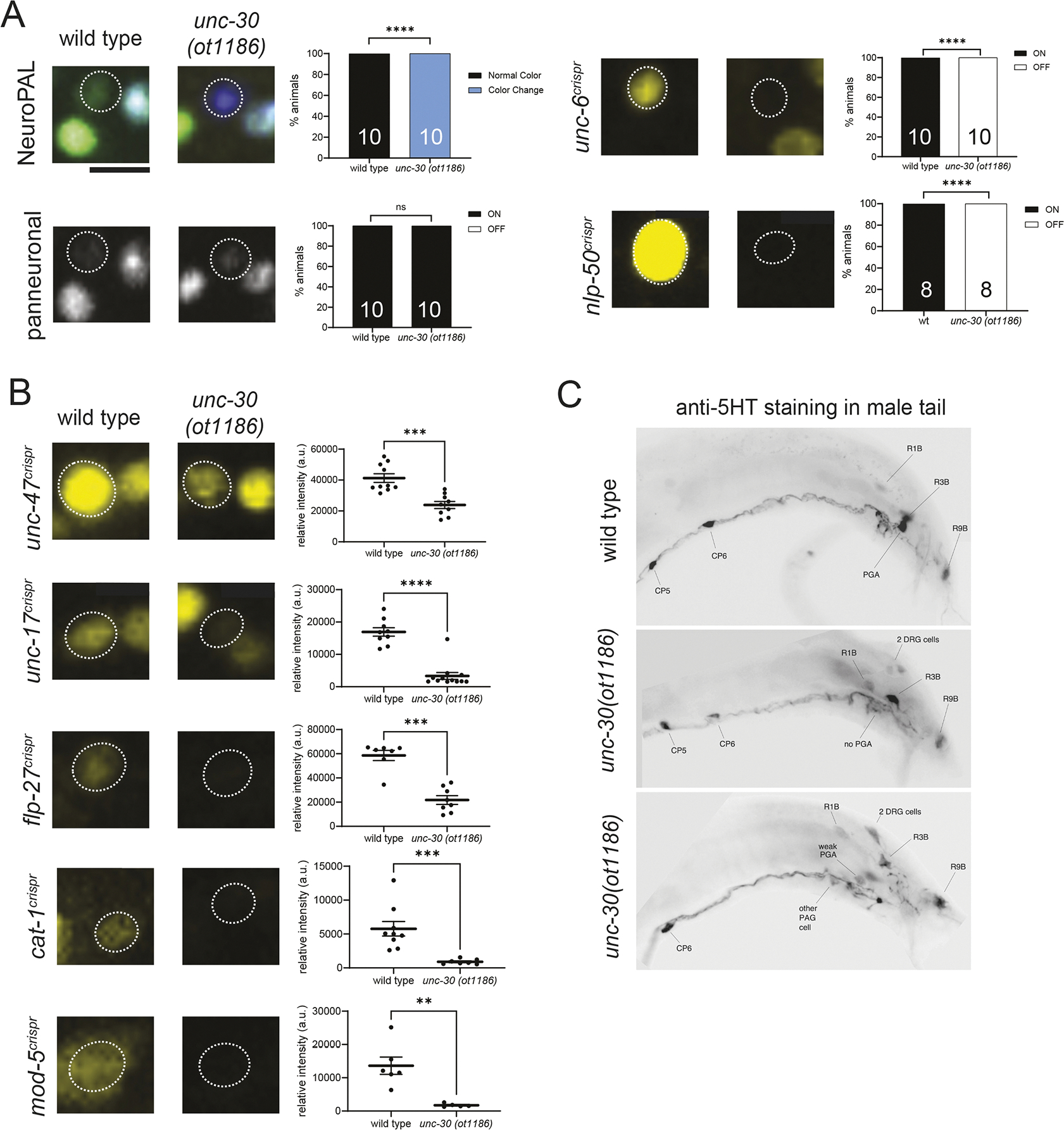
The *unc-30* Pitx-type homeobox gene controls differentiation of the PGA neuron. (A) In *unc-30(ot1186)* null mutant animals, the NeuroPAL (*otIs669*) color code of PGA is changed from orange to blue. Expression of *unc-6(syb5064)* and *nlp-50(syb2704)* in PGA is abolished in *unc-30(ot1186)* null mutants. The number of animals scored is shown within each bar. ****P≤0.0001 (Fisher’s exact test). ns. not significant. (B) *unc-30(ot1186)* null mutants show decreased expression of several PGA markers, including *unc-47(syb7566)*, *unc-17(syb4491)*, *flp-27(syb4413)*, *cat-1(syb6486)* and *mod-5(vlc47).* Error bars indicate s.e.m. ****P≤0.0001, ***P≤0.001, **P≤0.01 (Mann–Whitney test). (C) Loss of anti-serotonin antibody staining in *unc-30(ot1186)* null mutant animals. 0/52 male tails had strong PGA staining; 3/15 had a weakly staining PGA (bottom panel). PAG, preanal ganglion.

To assess *unc-30* function, we engineered a molecular null allele, *ot1186*, that eliminates the entire coding region of the locus using CRISPR/Cas9 genome engineering (Leyva-Diaz and Hobert, 2022). We found that the entire cohort of neurotransmitter and neuropeptide reporters (*unc-17*, *unc-47*, *mod-5*, *cat-1*, *flp-27*, *nlp-50*) fails to be properly expressed in the PGA neuron of these *unc-30* null mutants (Fig. 5A,B). Moreover, *mod-5*-dependent 5-HT antibody staining was absent in the PGA neuron of *unc-30* mutants (Fig. 5C). *unc-6* reporter allele expression was also affected, as was the normal color code of NeuroPAL (Fig. 5A) (see Materials and Methods for general notes on the usage of NeuroPAL). However, since the pan-neuronal marker of the NeuroPAL transgene was not affected in PGA, we can conclude that PGA is generated in *unc-30(ot1186)* mutant animals but fails to adopt its proper identity. *unc-30* therefore can be classified as a master-regulatory terminal selector of PGA identity, mirroring its terminal selector function in several sex-shared neuron classes previously examined in the hermaphrodite, namely the D-type motor neuron, the PVP and the AVJ neuron classes (Cinar et al., 2005; Jin et al., 1994; Pereira et al., 2015; Reilly et al., 2022). Intriguingly, the NeuroPAL transgene generates a novel mTagBFP2 signal in PGA in *unc-30(ot1186)* animals (Fig. 5A), indicating that PGA may have undergone an identity transformation to another neuron class. We cannot presently tell what this alternative neuronal identity may be since the NeuroPAL transgene drives TagBFP from 12 different promoters, expressed in ten different neuron classes (Tekieli et al., 2021).

### The Prop1 homolog *unc-42* and the Otx homolog *ttx-1* control different aspects of MCM neuron differentiation

**Fig. 6.**
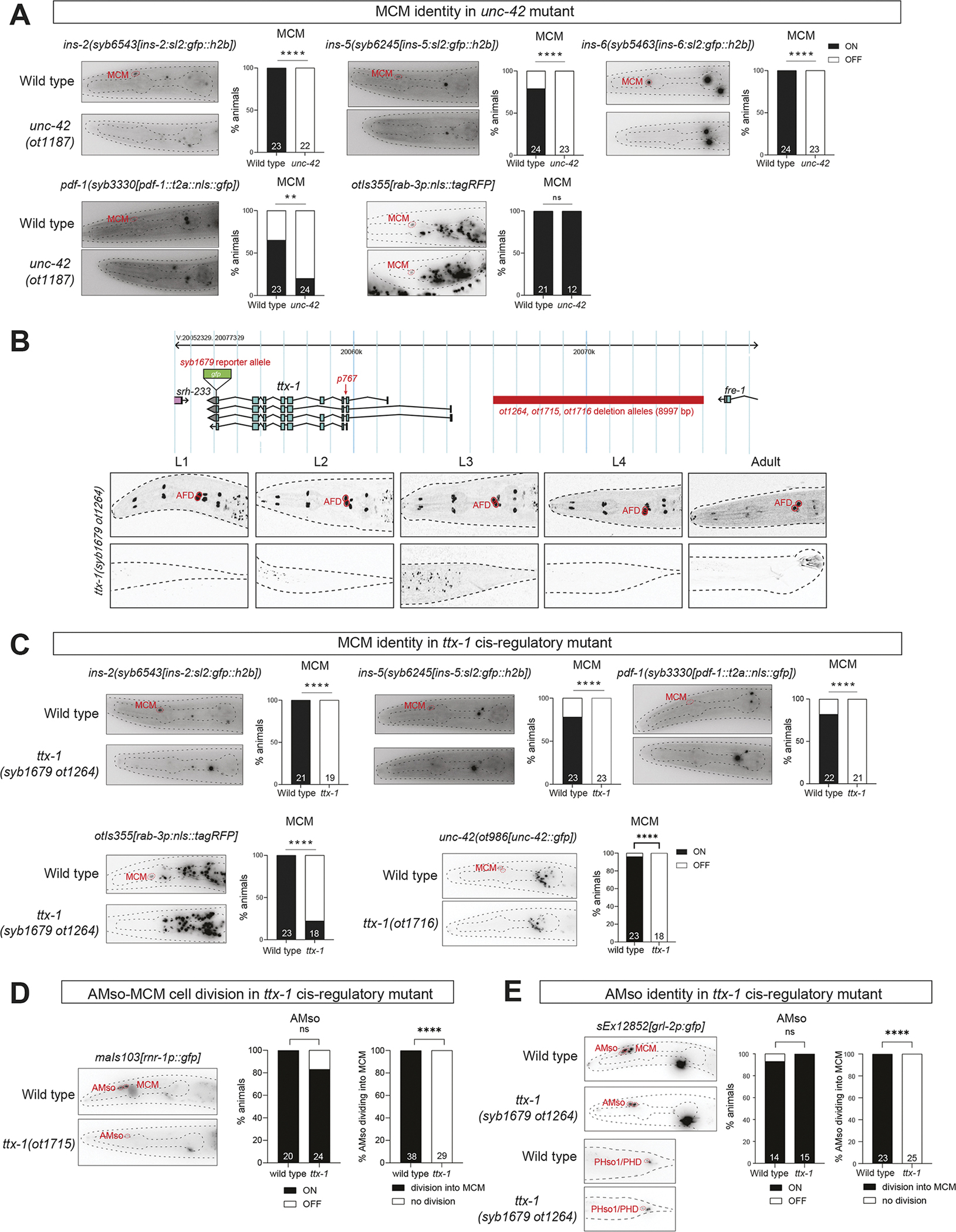
Effect of the Otx-type *ttx-1* and Prop-type *unc-42* homeobox genes on MCM neuron differentiation. (A) MCM terminal identity markers, but not a pan-neuronal marker, are lost in *unc-42(ot1187)* null mutants. Grayscale images are representative of expression of four terminal identity markers [*ins-2(syb6543)*, *ins-5(syb6245)*, *ins-6(syb5463)* and *pdf-1(syb3330)*] and one pan-neuronal marker (*rab-3* array *otIs355*) in a wild-type (control) and *unc-42(ot1187)* null mutant background. Dashed lines are used to trace the contours of the animal and its pharynx. Bar graphs show the percentage of animals that retained or lost expression in MCM. (B) A *gfp*-tagged, *cis*-regulatory allele of *ttx-1*, *syb1679 ot1264*, results in loss of expression in male-specific neurons MCM and PHD. Diagram of the *ttx-1* locus showing the extent of the *ot1264* deletion (red bar) and the *syb1679* edit which inserts a *gfp* in-frame with *ttx-1* for visualizing the protein expression. Representative images show that in an *ot1264* deletion background, *ttx-1* is not expressed in MCM and PHD. Dashed lines are used to trace the contours of the animal. (C) MCM terminal identity markers and a pan-neural marker, are lost in *ttx-1(syb1679 ot1264) cis*-regulatory mutants. Grayscale images are representative of expression of three terminal identity markers [*ins-2(syb6543)*, *ins-5(syb6245)* and *pdf-1(syb3330)* reporter alleles] and one pan-neuronal marker (*rab-3* reporter array *otIs355*) in a wild-type (control) and *ttx-1(syb1679 ot1264)* mutant background. *unc-42(ot986)* expression is also abolished in *ttx-1(ot1716)* animals, an allele molecularly identical to *ttx-1(ot1264)*. Dashed lines are used to trace the contours of the animal and its pharynx. The bright extra signal between the first and second bulb of the pharynx in *ttx-1(syb1679 ot1264)* mutant animals is likely TTX-1::GFP (see B, *syb1679*) signal coming from AFD. The *ot1264* regulatory allele does not disrupt *ttx-1* expression in a number of cells, including AFD (Reilly et al., 2022). Bar graphs show the percentage of animals that retained or lost expression in MCM. (D) The S-phase marker *maIs103(rnr-1p::gfp)* reveals impaired AMso-MCM cell division in animals carrying *ttx-1(ot1715)*, an allele molecularly identical to *ttx-1(ot1264).* The left bar graph shows the percentage of animals in which *maIs103* is expressed in AMso, while the right bar graph shows the percentage of individual AMso cells that divide into MCM. (E) *ttx-1(syb1679 ot1264)* animals show impaired MCM differentiation from AMso glia. Grayscale images are representative of expression of *grl-2* (reporter array *sEx12853*), an AMso terminal identity marker in a wild-type (control) and *ttx-1(syb1679 ot1264)* mutant background. Dashed lines are used to trace the contours of the animal and its pharynx. The left bar graph shows the percentage of animals that retained or lost *grl-2* (reporter array *sEx12853*) expression in AMso. The right bar graph shows the percentage of AMso that gave rise to MCM. During AMso to MCM transdifferentiation in wild-type animals, the GFP protein from *grl-2*-expressing AMso perdures into MCM as it divides (Sammut et al., 2015). In *ttx-1(syb1679 ot1264)* mutants, no perdurance is seen. The lower panel shows *grl-2* (reporter array *sEx12853*) expression in PHso1/PHD is unaffected by *ttx-1(syb1679 ot1264).* **P≤0.01, ****P≤0.0001 (Fisher’s exact test). ns, not significant.

We discovered a similar terminal selector role for the Prop1 homolog *unc-42* homeobox gene, which, like *unc-30*, is expressed in a single male-specific neuron class, the peptidergic MCM neuron in the head of the worm, as it transdifferentiates from the AMso cell (Fig. 3B). Our analysis of neuropeptide reporter alleles defined several markers for MCM, including reporter alleles for *pdf-1*, *ins-2*, *ins-5* and *ins-6.* We found that all four markers fail to be properly expressed in the MCM neurons of *unc-42* null mutant animals, containing an entire deletion of the *unc-42* coding region, generated by CRISPR/Cas9 genome engineering (Fig. 6A; Materials and Methods). Expression of the pan-neuronal marker *rab-3* was unaffected in *unc-42* null mutant animals, suggesting that MCM neurons are properly generated (i.e. transdifferentiated from the AMso amphid socket glia), but fail to adopt their unique identity (Fig. 6A). We conclude that *unc-42* acts as a terminal selector of MCM identity.

Since homeodomain proteins are known to act in combinations, we also tested the role of another homeobox gene that we found to be expressed in MCM during their initial differentiation and throughout adulthood, the Otx-type homeobox gene *ttx-1* (Fig. 3B). To analyze *ttx-1* function, we could not utilize a molecular null allele of the entire locus since such a deletion results in embryonic lethality (Reilly et al., 2022). In a previous study on *ttx-1* function in the hermaphrodite nervous system, we had circumvented this problem by generating a *cis*-regulatory allele, *ot1264*, a 9 kb deletion of an enhancer region that resulted in loss of *ttx-1* expression in several sex-shared neurons, but not in other tissues in which *ttx-1* function is required for embryonic viability (Reilly et al., 2022). Using a *gfp*-tagged *ttx-1* reporter allele, we examined whether this *cis*-regulatory allele also eliminates expression of *ttx-1* from the male-specific neuron classes in which *ttx-1* is normally expressed. We found this to indeed be the case (Fig. 6B). Using this *cis*-regulatory allele, we found that elimination of *ttx-1* displays the same phenotype as *unc-42* null mutant animals: expression of *pdf-1*, *ins-2* and *ins-5* is lost (Fig. 6C). In this case, however, we also failed to detect expression of both the pan-neuronal marker *rab-3* and the terminal selector *unc-42*, raising the possibility that the MCM neurons may not be properly generated at all (Fig. 6C). Consistent with this notion, an S-phase cell division marker, which normally marks the division that generated MCM (Sammut et al., 2015), failed to show its normal pattern of onset in *ttx-1* mutants (Fig. 6D).

Since *ttx-1* is, in contrast to *unc-42*, already expressed in the AMso glial cell from which the MCM neuron transdifferentiated (Fig. 3B), we assessed AMso glia (as well as PHso1) differentiation defects in *ttx-1(syb1679 ot1264)* mutants. We found no effect on the expression of the *grl-2* glia marker in juvenile AMso and PHso1 glia (Fig. 6E). However, we noted that after the cell division of the embryonically generated AMso during sexual maturation in larval stage animals, which generates the adult AMso and the MCM neurons, the expression of *grl-2* from embryonically generated AMso perdures in the MCM neurons (Sammut et al., 2015). We failed to detect such perdurance in *ttx-1(syb1679 ot1264)* mutants (Fig. 6E), again consistent with a failure of AMso to properly divide. We conclude that *ttx-1* does not control overt AMso differentiation per se, but rather ensures its ability to divide and transdifferentiate into the MCM neuron.

### The LIM homeobox gene *lim-6* affects DVE and DVF differentiation

**Fig. 7.**
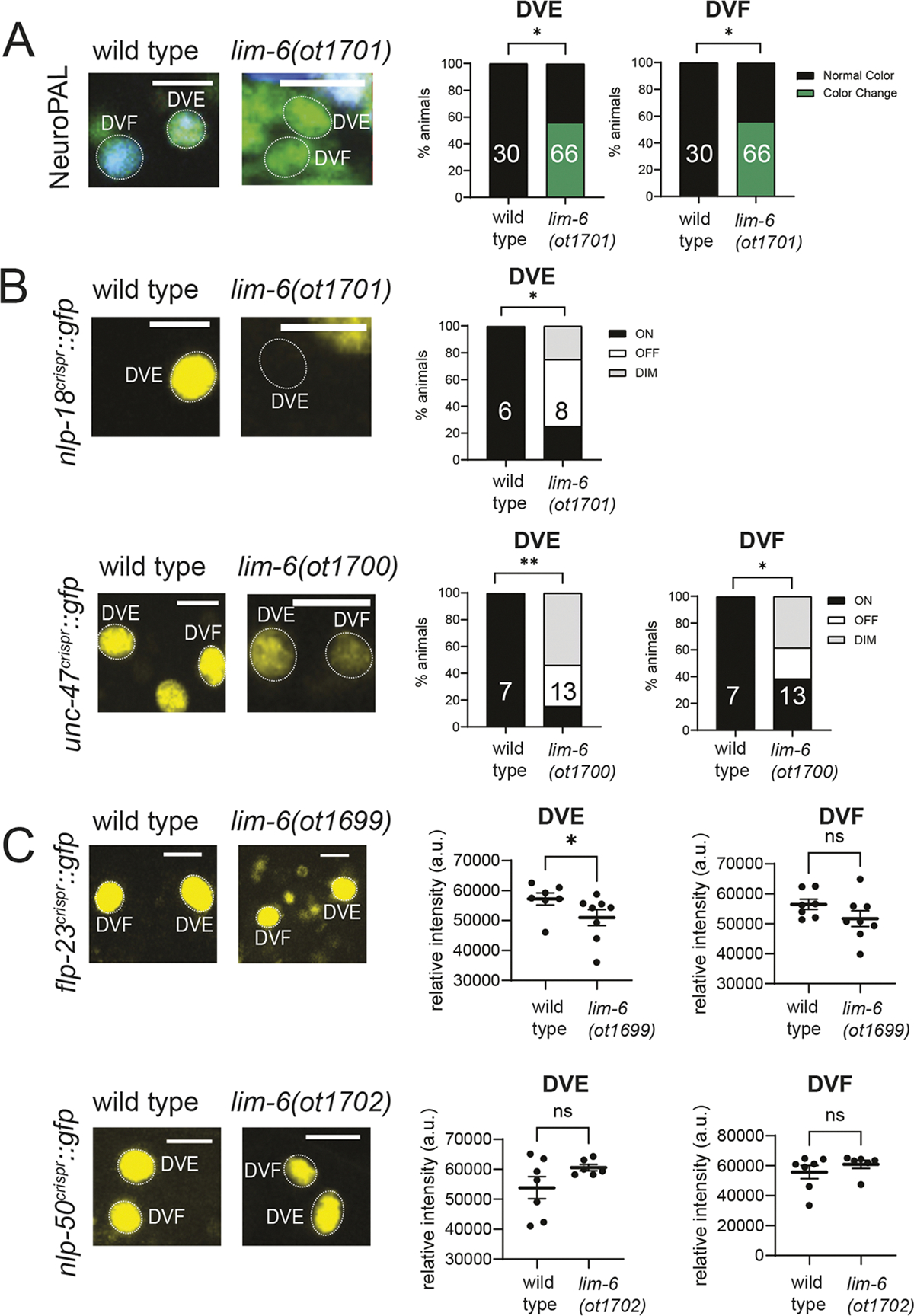
The Lmx-type *lim-6* homeobox gene affects differentiation of DVE and DVF neurons. (A) In nearly half of *lim-6(ot1701)* null mutant animals, the NeuroPAL (*otIs696*) colors of DVE and DVF are changed from turquoise to green, suggesting loss of blue-expressing reporters. Due to low mating efficiency of *lim-6* null mutants, molecularly identical null deletions were generated in the background of the markers in B and C by CRISPR/Cas9-engineering using the same guide RNAs and repair templates (see Materials and Methods). These alleles are *lim-6(ot1699)*, *lim-6(ot1700)*, *lim-6(ot1701)* and *lim-6(ot1702)*. (B) *lim-6(ot1701)* and *lim-6(ot1700)* null mutants display defects in the expression of DVE and DVF markers *nlp-18(ot1421)* and *unc-47(syb7566)*. (C) *lim-6(ot1699)* and *lim-6(ot1702)* null mutants do not show defects in the expression of the DVE and DVF markers *nlp-50(syb2704)* and *flp-23(syb3242).* Error bars indicate s.e.m. *P≤0.05, **P≤0.01 (A,B, Fischer’s exact test; C, Mann–Whitney). ns, not significant.

The LIM homeobox gene *lim-6*, the *C. elegans* ortholog of vertebrate Lmx1/2, is known to control the differentiation program of the sex-shared DVB neuron, located in the dorsorectal ganglion (Gendrel et al., 2016; Hobert et al., 1999). The expression of *lim-6* in two other, male-specific neurons of the dorsorectal ganglion, DVE and DVF, as well as the existence of cell markers for these two neurons, prompted us to assess the effect of complete, CRISPR/Cas9-mediated removal of the *lim-6* locus on the differentiation of these neurons. We found that the characteristic color code of the NeuroPAL transgene in the DVE and DVF neurons of *lim-6* null mutants is disrupted (Fig. 7A). DVE and DVF do not synthesize a currently known fast neurotransmitter system, but express the vesicular neurotransmitter transporter *unc-47* (Wang et al., 2024) and this expression was significantly affected upon loss of *lim-6* (Fig. 7B). Our neuropeptide expression analysis identified three neuropeptide reporter alleles expressed in these neurons, *flp-23* and *nlp-50* (expressed in both DVE and DVF), and *nlp-18* (expressed only in DVE) (Fig. 4). We found that expression of *nlp-18* in DVE was affected in *lim-6* null mutants (Fig. 7B), while there was a reduction in the expression levels of *flp-23* in DVE but not DVF. However, expression of *nlp-50* in both neurons was not affected in *lim-6* null mutants (Fig. 7C). We conclude that *lim-6* is required for the proper differentiation of DVE and DVF.

The DVE and DVF neurons also co-express the Eve homolog *vab-7*. We found that in *vab-7* mutants marker gene expression in neurons of the dorsorectal ganglion do not appear to be affected, but we noted an increased number of neurons in the ganglion, indicating lineage division defects, which we did not pursue further.

### The LIM homeobox gene *lin-11* impacts differentiation of several neuron classes

**Fig. 8.**
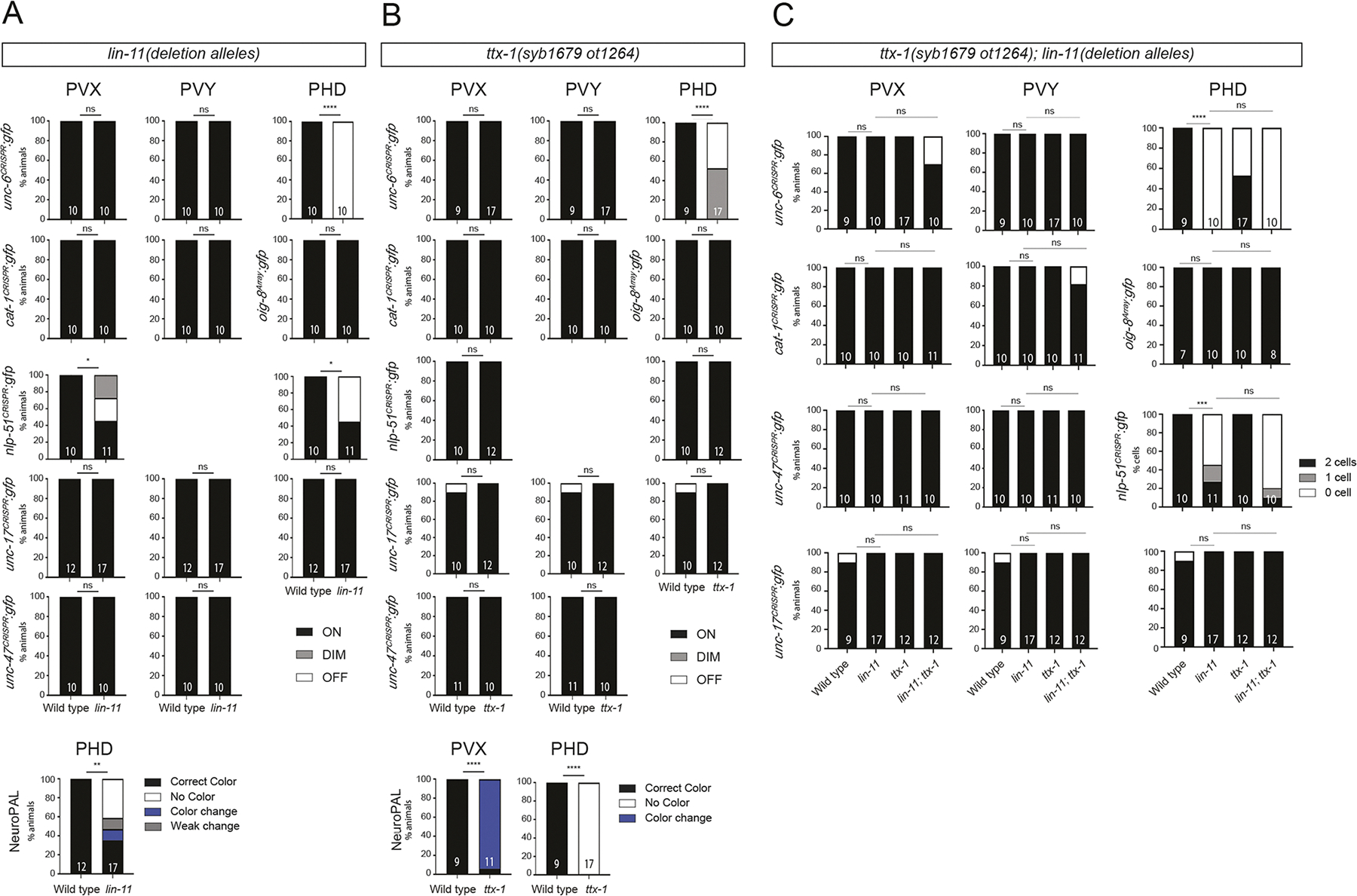
Effect of the LIM-type homeobox gene *lin-11* and the Otx-type *ttx-1* homeobox gene on PVX, PVY and PHD differentiation. (A) Bar graphs showing the percentage of animals that retained or lost signal for six terminal identity markers [*unc-6(syb5064)*, *cat-1(syb6486)*, *oig-8* array (*drpIs4*), *nlp-51(syb3936)*, *unc-17(syb4491)*, *unc-47(syb7566)*] as well as quantifying the NeuroPAL(*otIs669*) color in the male-specific neurons (PVX, PVY and PHD) of wild type and *ttx-1(syb1679 ot1264)* regulatory mutants. (B) Bar graphs showing the percentage of animals that retained or lost signal for six terminal identity markers [*unc-6(syb5064)*, *cat-1(syb6486)*, *oig-8* array (*drpIs4*), *nlp-51(syb3936)*, *unc-17(syb4491)*, *unc-47(syb7566)*] as well as quantifying the NeuroPAL(*otIs669*) color in the male-specific neurons (PVX, PVY and PHD) of wild type and *lin-11* null mutants [*lin-11(ot1483);unc-6(syb5064)*, *lin-11(ot1472);cat-1(syb6486)*, *lin-11(ot1521);oig-8* array (*drpIs4*), *lin-11(ot1444);nlp-51(syb3936)*, *lin-11(ot1026);unc-17(syb4491)*, *lin-11(ot1497); unc-47(syb7566)*]. (C) Bar graphs showing the percentage of animals that retained or lost signal for six terminal identity markers [*unc-6(syb5064)*, *cat-1(syb6486)*, *oig-8* array (*drpIs4*), *nlp-51(syb3936)*, *unc-17(syb4491)*, *unc-47(syb7566)*] in the male-specific neurons (PVX, PVY and PHD) of wild type and *ttx-1(syb1679 ot1264);lin-11* double mutants [*ttx-1(syb1679 ot1264);lin-11(ot1483);unc-6(syb5064)*, *ttx-1(syb1679 ot1264);lin-11(ot1472); cat-1(syb6486)*, *ttx-1(syb1679 ot1264);lin-11(ot1521);oig-8* array (*drpIs4*), *ttx-1(syb1679 ot1264);lin-11(ot1444);nlp-51(syb3936)*, *ttx-1(syb1679 ot1264); lin-11(ot1026);unc-17(syb4491)*, *ttx-1(syb1679 ot1264);lin-11(ot1497);unc-47(syb7566)*]. Using CRISPR/Cas9, multiple but genetically identical null alleles of *lin-11* were obtained (see Materials and Methods). *P≤0.05, **P≤0.01, ***P≤0.001, ****P≤0.0001 (Fisher’s exact test). ns, not significant.

We extended our homeobox mutant analysis to additional neuron types and found that CRISPR/Cas9-mediated removal of the entire coding region of the LIM homeobox gene *lin-11*, the *C. elegans* ortholog of vertebrate LHX1/5, has selective effects on the differentiation of several different male-specific neuron classes. The PHD neurons, generated during sexual maturation by transdifferentiation from PHso1 glia, were normally generated in *lin-11* null mutants and still expressed both their cholinergic identity (*unc-17*), as well as the single Ig domain protein *oig-8* (Fig. 8A), a previously described marker of PHD identity (Howell and Hobert, 2017; Molina-Garcia et al., 2020). However, the NeuroPAL color code, as well as expression of the *nlp-51* neuropeptide and *unc-6*, were defective (Fig. 8A, Fig. S4A). The Otx-type homeobox gene *ttx-1*, which is also expressed in PHD, also affected *unc-6* expression and the NeuroPAL color code, but did not affect *nlp-51*, *oig-8* or PHD’s cholinergic identity (*unc-17*) (Fig. 8B, Fig. S4B). *lin-11; ttx-1* double mutants did not show more severe defects in PHD differentiation than each single mutant, i.e. neither *oig-8* nor *unc-17* markers were affected (Fig. 8C).

Besides PHD, the *lin-11* and *ttx-1* homeobox genes are also co-expressed in the cholinergic PVX and PVY interneurons. We did not observe any differentiation defects in PVX or PVY in *lin-11*, *ttx-1* or *lin-11; ttx-1* double mutants based on intact *unc-6* and *oig-8* expression, aminergic identity (*cat-1*), cholinergic identity (*unc-17*) and other terminal identity features (*unc-47*) (Fig. 8A–C). However, we found that in *lin-11* null mutant animals, but not in *ttx-1 cis*-regulatory mutants, the expression of *nlp-51* was affected in PVX neurons (Fig. 8A, Fig. S4A). *ttx-1* may nevertheless have a partial impact on PVX differentiation since we observed a change in the NeuroPAL color code (Fig. 8B, Fig. S4B). We conclude that, in male-specific tail neurons, *lin-11* and *ttx-1* are required to specify a small set of differentiation features of individual tail neuron classes.

**Fig. 9.**
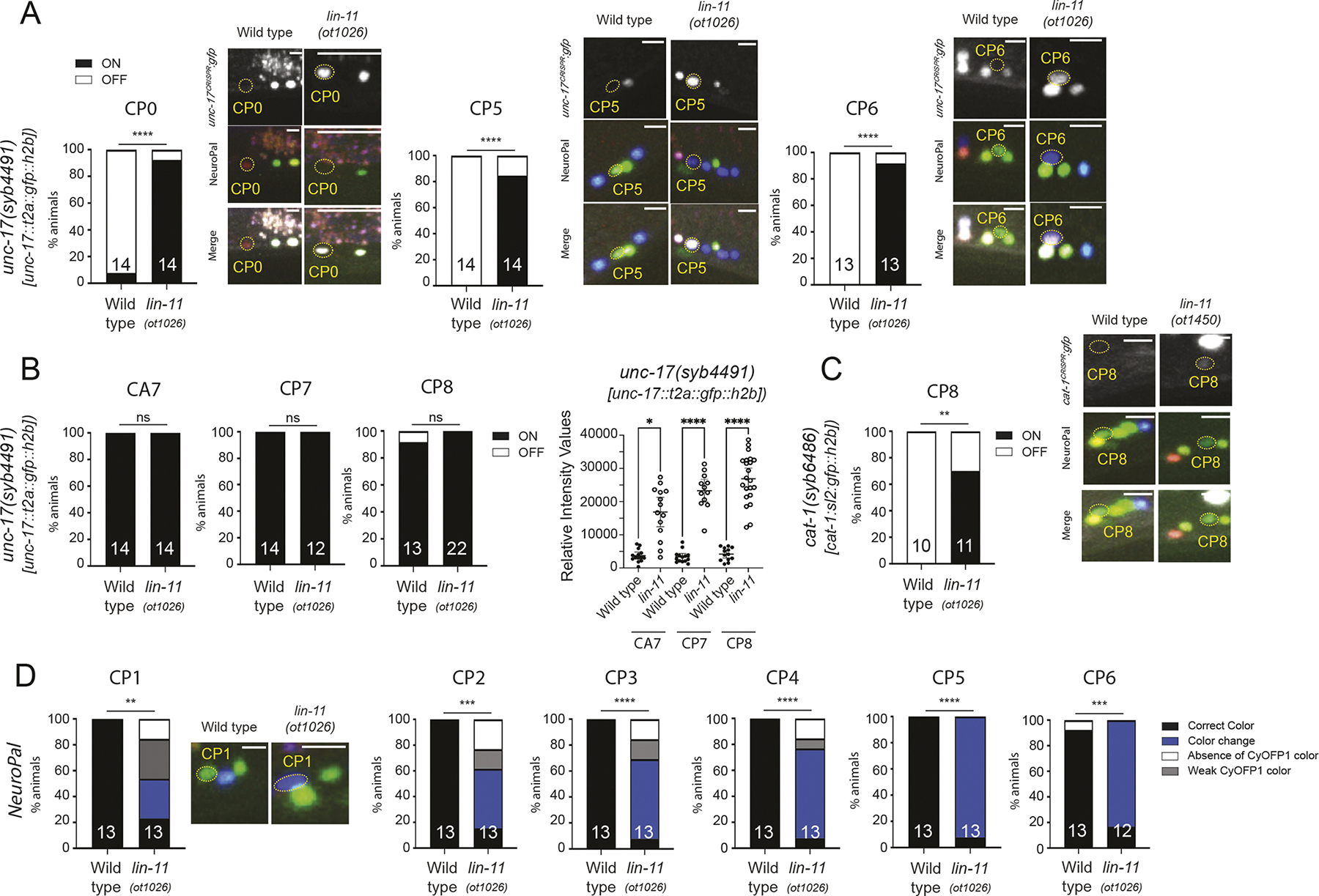
Effect of the LIM homeobox gene *lin-11* on CP neuron differentiation. (A) *unc-17*-positive CP neurons in *lin-11(ot1026)* null mutants. Representative images show the expression of *unc-17(syb4491)*, NeuroPAL*(otIs669)* landmark and their merge in wild type and *lin-11(ot1026)* mutants. Bar graphs show the percentage of animals expressing ectopic *unc-17*. (B) Absence of *lin-11* increases the expression of *unc-17* in several CP neurons. Bar graphs show the percentage of animals expressing *unc-17* as well as intensity values in wild type and *lin-11(ot1026)* null mutants. (C) *lin-11* null mutants show ectopic *cat-1* expression in CP8 male-specific neurons. Representative images show the expression of *cat-1(syb6486)*, NeuroPAL*(otIs669)* landmark and their merge in wild type and *lin-11(ot1450)* mutants. Bar graph shows the percentage of animals expressing ectopic *cat-1*. (D) *lin-11(ot1026)* mutants show alter NeuroPAL colors in CP neurons. Representative images show an overlay of the NeuroPAL*(otIs669)* pseudocolors in wild type and *lin-11(ot1026)* null mutants. CP neurons have a stronger blue fluorophore signal in *lin-11(ot1026)* null mutants compared to wild type. Bar graphs show the percentage of animals with no color change (Correct Color), a color change (Color Change), absence of the orange color (Absence of CyOFP1 color) and a dim orange color (Weak CyOFP1 color). *P≤0.05, **P≤0.01, ***P≤0.001, ****P≤0.0001 (Fisher’s exact test and unpaired, two-tailed Student’s *t*-test for quantification of relative intensity values). ns, not significant.

In the ventral nerve cord, *lin-11* is expressed in a subset of CA and all CP neurons. Each of these neuron classes falls into distinct subtypes based on neurotransmitter expression (Wang et al., 2024), neuropeptide expression (Table 2) and color codes of the NeuroPAL transgene (Tekieli et al., 2021). We found that cholinergic identity (*unc-17* expression) of the CA1–4 neurons, which normally express *lin-11*, was unaffected in *lin-11* null mutants (13/14 animals showed normal expression of an *unc-17* reporter allele). Instead, several of the CP neurons (CP0, CP5, CP6, CP7, CP8), as well as CA7, which are either not cholinergic or express *unc-17* only very weakly, expressed *unc-17* either ectopically or much more strongly in *lin-11* null mutants (Fig. 9A,B). We also observed ectopic expression of *cat-1* in CP8 (Fig. 9C), as well as a concomitant novel NeuroPAL color code (gain of blue color) in the CP1–6 neurons (Fig. 9D). Since the CA neurons, which are sisters of the CP neurons, are cholinergic (Wang et al., 2024), a CP-to-CA identity switch could be envisioned. However, such transformation is unlikely since, first, the change in NeuroPAL color code is not consistent with such a switch (NeuroPAL did not mark the CA neurons with any signal beyond the pan-neuronal signal, yet there was a gain in orange signal in *lin-11* mutants) and, second, we found 5-HT antibody staining of CP neurons to be unaffected in *lin-11* mutants (52/55 animals show normal 5-HT staining), indicating the CPs do retain aspects of their original identity. At this point, we can only infer that in *lin-11* mutants the CP neurons are not properly specified.

### Behavioral deficits of *lin-11* and *ttx-1* mutants

**Fig. 10.**
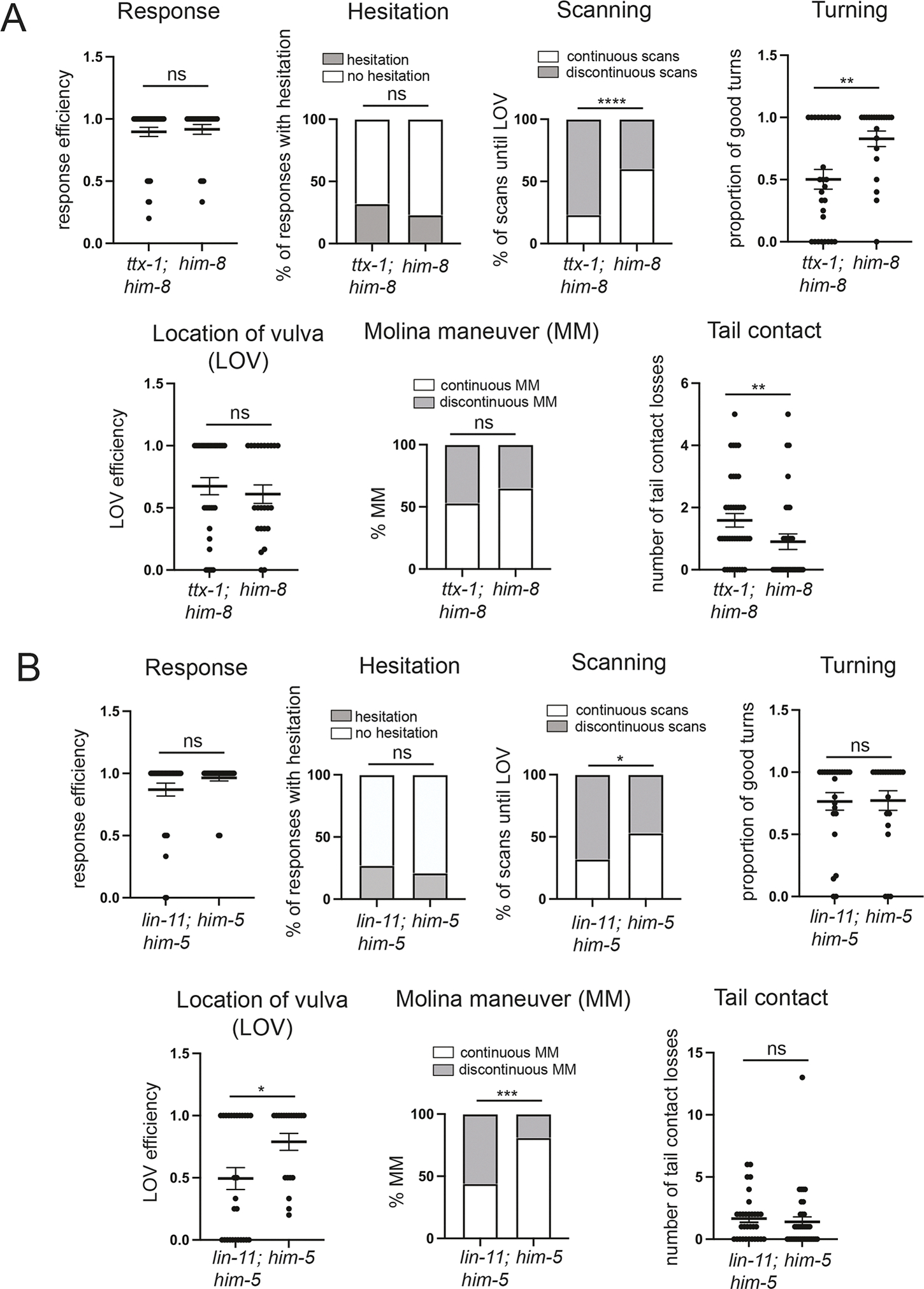
*lin-11* and *ttx-1* mutants display male mating defects. (A) Male mating steps in *ttx-1(syb1679 ot1264); him-8(e1489) IV* mutant males, mated with *unc-51* mutant hermaphrodites. See Materials and Methods for details on scored behaviors. Mann–Whitney tests were used to compare efficiencies of response, turning, location of vulva and tail contact of control and mutant males; χ^2^ was used to compare the proportion of responses with hesitation and proportion of continuous scans and Molina Maneuvers (MM). ns, not significantly different (*P*≥0.05). **P≤0.01, ****P≤0.0001. *n*=number of events; 23 *ttx-1; him-8* and 28 *him-8* males were scored. (B) Male mating steps in *lin-11(n389) I; him-5(e1467)* mutant males, mated with *unc-51* mutant hermaphrodites. See Materials and Methods for details on scored behavior. Mann–Whitney test was used to compare efficiencies of response, turning, location of vulva and tail contact of control and mutant males; χ^2^ was used to compare the proportion of responses with hesitation and proportion of continuous scans and Molina Maneuvers. ns, not significantly different (*P*≥0.05). *P≤0.05, ***P≤0.001. *n*=number of events; 18 *lin-11; him-5* and 20 *him-5* were scored.

Owing to their strong locomotory defects, we were not able to assess the behavioral consequences of loss of MCM differentiation in *unc-42* null mutants or loss of PGA differentiation in *unc-30* null mutants. However, superficially normal locomotion of *lin-11(n389)* animals and *ttx-1(syb1679 ot1264)* animals did allow us to assess their impact on male mating behavior. The *lin-11*- and *ttx-1*-expressing PVX and PVY interneurons have been shown to be involved in the male’s contact-based scanning of the hermaphrodite’s surface for the vulva (Sherlekar et al., 2013). This scanning behavior also requires the *lin-11*- and *ttx-1*-expressing PHD neuron, which is presynaptic to both PVX and PVY (Molina-Garcia et al., 2020). We found that both tail contact and scanning behavior of *ttx-1 cis*-regulatory mutant males is defective (Fig. 10A). Scanning behavior, but not tail contact, was also defective in *lin-11* mutant males (Fig. 10B). PHD is also involved in another aspect of the mating behavior, the Molina maneuver, a re-engagement process of males with a mate after an unsuccessful mating attempt (Molina-Garcia et al., 2020). Molina maneuvers were defective in *lin-11* mutant males, but not in *ttx-1 cis*-regulatory mutants, consistent with *lin-11* mutant males appearing to have a more severe PHD differentiation defect than *ttx-1 cis*-regulatory mutant males (Fig. 8).

While other aspects of male mating behavior are intact in *ttx-1* and *lin-11* mutants, we also noted two defects that were not predicted by their expression pattern (Fig. 10A,B): *lin-11* mutants displayed defects in vulval stop behavior and *ttx-1 cis*-regulatory mutants displayed defects in turning behavior. Whether these defects are also the result of PVX, PVY or PHD differentiation defects remains unknown.

## DISCUSSION

### The male-specific nervous system of *C. elegans*

The nervous system of male nematodes is substantially larger than the nervous system of hermaphrodites, owing largely to sex-specific neuroblast proliferation that generates a wide spectrum of distinct neuron types only in males (Sulston et al., 1980). While brain size differences are also apparent in different areas of human male and female brains (DeCasien et al., 2022), the underlying cellular basis of such differences remains unclear. While the number, lineage and morphology of male-specific neurons in *C. elegans* have been precisely delineated (Cook et al., 2019; Jarrell et al., 2012; Sulston et al., 1980), mechanisms that specify the unique identities of these male-specific neurons have mostly been explored in the context of developmental patterning, namely of (1) the ray sensory neuron classes, a prominent subgroup of male-specific neurons (Lints and Emmons, 1999; Lints et al., 2004; Siehr et al., 2011; Yang et al., 2007), (2) two male-specific neuron classes that are generated by transdifferentiation (MCM, PHD) (Molina-Garcia et al., 2020; Sammut et al., 2015), (3) one male-specific neuron class for which sex specificity is the result of sex-specific cell death (CEM) (Peden et al., 2007; Schwartz and Horvitz, 2007; Shaham and Bargmann, 2002), and (4) several male-specific motor and interneurons in the ventral cord (CA,CP) (Kalis et al., 2014, 2022). Earlier patterning information is available for the developmental specification programs of other male-specific neurons (Chisholm, 1991; Yu et al., 2003), yet information about terminal differentiation programs had been limited. We have begun here to rectify these limitations, by (1) establishing a protein expression atlas of a family of transcription factors, homeodomain proteins, and (2) defining the role of these proteins in cell fate specification using a new set of reagents that allowed us to define cell identity.

### Homeobox gene expression

In the hermaphrodite, an analysis of homeodomain protein expression has shown that each individual terminally differentiated neuron class can be defined by a unique combination of homeodomain proteins (Reilly et al., 2020). Even though we analyzed only half of all conserved *C. elegans* homeodomain proteins in this study, homeodomain proteins again emerge as accurate descriptors of neuronal identity in the male-specific nervous system. The expression of homeobox genes also delineates neuronal subclasses within the cardinal 25 male neuron classes. Such subclassification has been evident already based on terminal differentiation markers (e.g. among CA, CP or ray neurons), but for other classes, such subclassification was not previously appreciated (EF, DX neurons). We assigned at least one homeodomain protein to all 25 cardinal neuron classes of the male-specific nervous system of *C. elegans*, and, except for two closely related groups of neuron classes, we observed neuron-specific combination of homeodomain proteins in each of these classes. The broad coverage of neuron classes with a limited number of examined homeodomain proteins (40 out of 80 conserved homeodomain proteins) predicts that a complete map of all homeodomain proteins will result in neuron type-specific combination of homeodomain proteins for each individual male-specific neuron class, as observed in the hermaphrodite nervous system.

As in the sex-shared nervous system, there is no obvious match of a specific neurotransmitter system with a specific homeobox signature, at least as far as the most commonly deployed neurotransmitter systems (acetylcholine, glutamate, GABA) are concerned, thereby corroborating the theme that neurotransmitter identity of distinct neuron classes is controlled in a cell type-specific manner through distinct regulatory factors. This notion is further supported by the dissection of the *cis*-regulatory architecture of the *unc-17* and *eat-4/VGluT* locus, which identified distinct *cis*-regulatory elements driving gene expression in distinct male-specific neuron classes (Serrano-Saiz et al., 2020).

The HOX cluster homeodomain proteins, which constitute only six of the 102 homeodomain proteins in *C. elegans*, stand out in several regards. First, unlike in head ganglia, HOX gene expression very densely covers almost all neuron classes in male tail ganglia, oftentimes in an overlapping manner (summarized in Fig. 2). This is a mirror image of many non-cluster homeobox genes that are expressed in multiple sex-shared head neuron classes but not at all in tail ganglia (Table S1). Second, HOX cluster genes show clear anterior/posterior patterns of spatial co-linearity within tail ganglia. Such co-linearity had already been observed in ventral cord neurons in *C. elegans* of sex-shared and sex-specific neurons (Kalis et al., 2014; Kratsios et al., 2017; Smith et al., 2024). Our systematic analysis of all HOX cluster genes extends such anterior/posterior-patterned expression throughout all neurons within male tail ganglia (i.e. the much-expanded lumbar ganglion in males, as well as the male-specific cloacal ganglion). This neuronal expression is – like all homeodomain protein expression patterns that we describe here – maintained throughout mature, adult stages.

### Homeobox gene function

Previous work has defined functions of HOX cluster genes in the male nervous system (Lints and Emmons, 1999; Lints et al., 2004; Tekieli et al., 2021). We have leveraged here the power of the NeuroPAL cell fate tool, in combination with additional cell fate markers that we developed, to describe the impact of removal of non-HOX cluster homeobox genes on the differentiation program of individual male-specific neurons. In two cases, notably PGA and MCM, we have shown that, of the many neuron type-specific identity features that we tested, every single feature is affected in the respective homeobox gene mutant (*unc-30* for PGA; *unc-42* for MCM), arguing for a strict co-regulation of distinct identity markers, the key defining feature of a terminal selector-type regulatory logic (Hobert, 2016). In the case of MCM, we have also identified a homeobox gene, *ttx-1*, which appears to act at an earlier step in the differentiation program by apparently priming the AMso glial cell to transdifferentiate into MCM.

We had previously noted that a subset of terminal selectors demarcate neurons that are more highly interconnected with each other compared to other neurons and suggested that such terminal selectors may act as ‘circuit organizers’ (Berghoff et al., 2021). The best described of these cases is the *unc-42* homeobox gene, which acts as a terminal selector in all 15 interconnected neurons in a nociceptive reflex circuit (Berghoff et al., 2021). The function of *unc-42* as a terminal selector in the male-specific MCM neuron, the only sex-specific neuron in which *unc-42* is expressed, fits into this theme since MCM is extensively connected to this *unc-42(+)* reflex circuit (Cook et al., 2019). We therefore predict that in *unc-42* mutants MCM may not only lose features of its molecular identity but may also fail to wire into the ‘*unc-42* circuit’. It is conceivable that UNC-42 may regulate the expression of cell surface molecules that allow for selective fasciculation of these neurons.

We have also described cases here in which it is not clear whether a homeobox gene acts as a master-regulatory terminal selector. In the DVE and DVF neurons, *lim-6* appears to control many, but not all, identity features. The effect of *lin-11* and *ttx-1* in the synaptically interconnected PVX, PVY and PHD neurons is restricted to only a small number of identity features. Nevertheless, these effects appear to have significant functional consequences, as evidenced by the behavioral defects observed in *ttx-1* and *lin-11* mutants. *lin-11* and *ttx-1* may either act redundantly with other homeobox genes to specify PVX, PVY and PHD differentiation (e.g. HOX cluster genes) and/or other PVX/PVY/PHD-expressed factors may fulfill a master regulatory terminal selector role. For the P-neuroblast-derived PVX and PVY neurons, one intriguing candidate is the COE-type transcription factor UNC-3, which acts in combination with HOX cluster genes as a terminal selector of all sex-shared, P-neuroblast derived ventral cord cholinergic motor neurons (Feng et al., 2022; Kratsios et al., 2011). We find that PVX, PVY (as well as the cholinergic PVV and PDC) neurons co-express UNC-3 and HOX cluster genes (A.J.D. and O.H., unpublished), hinting towards their potential function as terminal selectors in these neurons as well.

Akin to the mating defects that we describe here for *lin-11* and *ttx-1* mutants, mating defects have also been observed in another homeobox gene, *mec-3* (Hodgkin, 1983; Iosilevskii et al., 2025). The sites of expression of *mec-3* that we described here match the function of the neurons and the specific behavioral defects in *mec-3* mutants (HOB neuron for vulva stopping; ray neurons for turning behavior) (Iosilevskii et al., 2025). Yet, as in the case of *lin-11* and *ttx-1* mutants, cell type-specific rescue experiments are needed to confirm that these genes do indeed act in these respective neurons to control animal behavior. Owing to pleiotropies of other homeobox gene mutants (e.g. Unc phenotype of *unc-42* and *unc-30*, Let phenotype of *ceh-43*, Clr phenotype of *ceh-10*, etc.), more cell type-specific knockout approaches will be needed to assess their impact on the function in the male-specific neurons that these genes are expressed in.

### Limitations and conclusions

There are several limitations of the work described here. The expression of half of all conserved homeodomain proteins still awaits investigation in the male-specific nervous system. Cell type-specific rescue experiments and/or cell type-specific knockout experiments are currently lacking and are particularly needed to link the behavioral defects of *lin-11* and *ttx-1* mutants to their specific cellular focus of action. Such approaches will require the development of drivers with the required cellular specificity. Despite these limitations, we can conclude that homeobox gene expression and function appears to be as pervasive in the sex-specific nervous system as it is in the sex-shared nervous system. These observations are in further support of the hypothesis that homeobox genes may have fulfilled an evolutionarily ancient function in neuronal cell type specification and diversification (Hobert, 2021).

## MATERIALS AND METHODS

### Strains

A list of strains used in this study is provided in Table S2.

### *C*. *elegans* genome-engineered strains

Many reporter alleles generated in this study were engineered by SunyBiotech using the CRISPR/Cas9 system to insert an SL2::GFP::H2B reporter cassette (indicated by the *syb* allele name in Table S2). Some of these strains have been recently described (Ma et al., 2021; Reilly et al., 2020, 2022; Ripoll-Sanchez et al., 2023); others were generated specifically for this study (Table S2).

All gene-deletion alleles were designed to remove the entire coding region of the gene, from start to stop codon, and include a null allele for *unc-42*, *ot1187*, generated by injecting the following crRNAs and repair template to completely remove the coding region of *unc-42*: crRNA (GCTCATtgtgtgagtgaaag), crRNA (tctcactgatagactaatgt), ssODN (ATCCCTTCAGAGCCATACTTCTCACTACTACCACCATCATAGAATCAAGACCTGAAATCGACCTAAAAAA). Two *ttx-1* alleles (*ot1715* and *ot1716*) molecularly identical to the previously published *ttx-1(ot1264)* (Reilly et al., 2022) were also generated for this study using the following crRNAs and repair templates: crRNA (TGCTCCACGGAGCCCAGAAG), crRNA (AGCTGATATGAACTGAGTTA), ssODN (GTTAGCTTACGGACAAATAGGTCACACTTCCCCTTCTCAGTTCATATCAGCTTTTGAAAAATGTGATAAA).

Owing to issues with their mating efficiency, multiple molecularly identical null alleles of *lin-11* and *lim*-6 were generated by injecting separate strains carrying reporter alleles for terminal identity markers, using the following crRNAs and repair templates: *lin-11* (*ot1026*, *ot1497*, *ot1444*, *ot1521*, *ot1472*, *ot1483*): crRNAs (attgagaagggagtaaaagg and CGTGGAATACTCCTGTATGT), ssODN (TTCGTGGTCGttcttcttcttcttctcctcctcctTACAGGAGTATTCCACGTTCGTGTAGTTTTTCTTC); *lim-6* (*ot1699*, *ot1700*, *ot1701*, *ot1702*): crRNAs (TGTGTTTTGTAGAAGACCGG and GAAAAGCAAAATAAAGCGGG), ssODN (gctcctgctctctctctctgtgttttgtag aagacgctttattttgcttttcacctcatattatttattt).

### Use of NeuroPAL

Sites of GFP reporter gene expression were determined using the NeuroPAL landmark strain (*otIs669* and *otIs696* transgenes) and male tail atlases previously described (Tekieli et al., 2021; Yemini et al., 2021). The identity of each neuronal type was identified by comparing the color, size and location of each neuron relative to one another. A detailed protocol to neuronal identification using NeuroPAL can be found at https://www.hobertlab.org/neuropal/.

We have also used NeuroPAL as a tool to record the proper execution of neuronal differentiation programs. The expression of specific combinations of the 41 reporter constructs present in the NeuroPAL transgene in an individual neuron class is a reflection of the proper execution of a neuronal differentiation program (Tekieli et al., 2021; Yemini et al., 2021). Hence, should the NeuroPAL color code change in a specific mutant background, overall changes in neuronal differentiation can be inferred because of the misregulation of a specific reporter construct located on the NeuroPAL transgene.

### Microscopy and mutant analysis

To prepare animals for imaging, a small agarose pad (5%) was cast on a standard imaging slide and worms were mounted and immobilized using a solution of 100 mM of sodium azide (NaN_3_). Images were acquired either using confocal laser scanning microscopes (Zeiss LSM880 and LSM980) or wide-field microscopy (Axio Imager Z2). Images were processed and analyzed using Zen (Zeiss) or Fiji (Schindelin et al., 2012) imaging software. All reporter reagents and mutants were imaged at 40× using fosmid or CRISPR reagents, unless otherwise specified.

For determining the expression pattern of homeobox genes or terminal identity markers, representative maximum intensity projections are displayed in grayscale, with gamma and histogram adjustments for visibility. For mutant functional analysis, representative maximum intensity projections are shown in inverted grayscale. In the case of all-or-nothing changes, a qualitative analysis using three qualifiers was used to score mutants: ON, the signal was still present in the mutant; OFF, the signal was lost; DIM, the signal was still present but drastically reduced. When changes were not all-or-nothing, a qualitative approach was used and the signal intensity was extracted using either Zen or Fiji.

To assay homeobox gene expression pattern at different larval and adult stages, animals were picked from a plate containing a mixed population of different stages. Different larval stages were differentiated according to size and well-known anatomical markers. Furthermore, animals were sexed under a high-magnification microscope and only male larvae were included in the analysis.

### Serotonin antibody staining

Anti-serotonin immunofluorescence was performed as previously described (Loer and Kenyon, 1993). Briefly, worms were fixed overnight in 1.5 ml microfuge tubes at 4°C in 4% paraformaldehyde in PBS; rinsed three times in PBSTx (0.5% Triton X-100/PBS), then incubated overnight at 37°C with gentle mixing in 5% β-mercaptoethanol in TrisTx (1% TX-100/0.1 M Tris, pH 7.4); rinsed twice in TrisTx, then once in collagenase buffer (1 mM CaCl_2_/TrisTx), then digested with 2000 Units/ml collagenase type IV (Sigma-Aldrich, C5138) in collagenase buffer until a few adult worms fragmented (typically 30–45 min); rinsed three times in PBSTx, incubated in 1% bovine serum albumin (BSA) in PBSTx for 1–2 h at room temperature, then incubated overnight at room temperature in 1:100 anti-serotonin (rabbit antiserum; Sigma-Aldrich, S5545) in 1% BSA/PBSTx; rinsed three to four times in 0.1% BSA/PBSTx for 1–2 h at room temperature, then incubated overnight at room temperature (in the dark) in 1:100 secondary antibody (goat anti-rabbit IgG, TRITC-conjugated; Sigma-Aldrich, T-2769); and rinsed three or four times in 0.1% BSA/PBSTx for 1–2 h at room temperature. Finally, worms were viewed and photographed with an Olympus BX60 upright fluorescence microscope equipped with a Magnafire CCD camera.

### Mating assays

Mating assays were performed on 9 cm NGM plates in which a bacterial lawn of 15 μl of OP50 was placed in the middle containing 30 *unc-51(e369)* hermaphrodites. Males were tested at day 1 of adulthood with 1-day-old *unc-51(e369)* hermaphrodites picked the night before as L4s. Each male was tested for 15 min. During this time, all steps of mating were scored in one or more hermaphrodites. Assays were replicated at least twice on different days and with different sets of males. Videos of mating events were recorded at 2 fps using LoopBio and visualized with QMPlay 2 to analyze the following steps of mating:

### Response

A male was scored as responding to mate contact if it placed its tail ventral side down on the hermaphrodite’s body and initiated the mating sequence by backing along the hermaphrodite’s body to make a turn. The response efficiency was calculated by dividing 1 (response) by the total number of contacts made with the mate before responding.

As a more sensitive measure of the quality of response, we scored hesitation during response. Hesitation is a switch in direction between forward and backward locomotion from the time the male establishes contact with the mate to the first turn (or to location of vulva if this occurs without the need of a turn).

### Scanning

A single scan was scored as the journey around the hermaphrodite’s body away from and returning to the vulva position. The first scan was counted as the journey from the point of first contact to the hermaphrodite vulva position. A scan was considered continuous if locomotion was maintained in the backward direction without switching direction or pausing (regardless of pause duration).

### Turning

Measured as proportion of good turns (number of good turns divided by total number of turns performed by the male) until location of vulva. A turn was considered good if it happened continuously while the worm was scanning backwards the tip of the hermaphrodite body to continue scanning the other side of the hermaphrodite without losing contact, switching direction or pausing before the turn.

### Location of vulva (LOV)

A male was considered successful in locating the vulva when they stopped scanning at the vulva position to try to insert the spicules. The LOV efficiency was calculated by dividing 1 (LOV) by the total number of times the male passed by the vulva without stopping there.

### Molina maneuvers

A continuous single maneuver was scored as the journey away from the vulva in forward locomotion, to a distance greater than two tail-tip lengths, and the return to the vulva in backward locomotion. Any visible pause during forward or backward locomotion was considered a STOP regardless of its duration. The category of discontinuous maneuver ‘switching’ was scored as a change in direction of locomotion while travelling away or towards the vulva without reaching it.

### Tail contact loss

The number of contact losses was scored as previously described (Sherlekar et al., 2013), i.e. the number of times that a male lost tail contact with the hermaphrodite during the mating trial (without counting male detachment after ejaculation).

## Supplementary Material

All Supp Figs and Tables

## Figures and Tables

**Table 1.** Summary of expression of 25 homeobox genes across the male *C. elegans* nervous system

Cardinal neuron class	Class member	Homeobox																				HOX cluster						
		*ceh-2*	*ceh-9*	*ceh-10*	*ceh-27*	*ceh-43*	*cog-1*	*dve-1*	*lin-11*	*ttx-1*	*unc-4*	*unc-30*	*unc-42*	*vab-3*	*vab-7*	*ceh-14*	*lim-6*	*lim-7*	*ceh-6*	*mec-3*	*unc-62*	*ceh-13*	*lin-39*	*mab-5*	*egl-5*	*nob-1*	*php-3*	
MCM	MCML/R																											4
CEM	CEM(D/V/L/R)																											1
CA	CA1																											4
	CA2																											5
	CA3																											5
	CA4																											5
	CA5																											3
	CA6																											3
	CA7																											4
	CA8																											4
	CA9																											4
CP	CP0																											5
	CP1																											4
	CP2																											4
	CP3																											4
	CP4																											4
	CP5																											5
	CP6																											4
	CP7																											3
	CP8																											3
	CP9																											4
HOA	HOA																											3
HOB	HOB																											4
PDC	PDC																											1
PGA	PGA																											6
PVV	PVV																											2
PVY	PVY																											5
PVX	PVX																											5
PVZ	PVZ																											3
DVE	DVE																											4
DVF	DVF																											6
DX	DX1/2																											3
	DX3/4																											1
EF	EF1/2																											3
	EF3/4																											2
RnA	R1A(L/R)																											2
	R2A(L/R)																											3
	R3A(L/R)																											2
	R4A(L/R)																											4
	R5A(L/R)																											5
	R6A(L/R)																											2
	R7A(L/R)																											3
	R8A(L/R)																											4
	R9A(L/R)																											3
RnB	R1B(L/R)																											2
	R2B(L/R)																											5
	R3B(L/R)																											3
	R4B(L/R)																											4
	R5B(L/R)																											2
	R6B(L/R)																											1
	R7B(L/R)																											3
	R8B(L/R)																											2
	R9B(L/R)																											1
PHD	PHD(L/R)																											6
PCA	PCA(L/R)																											2
PCB	PCB(L/R)																											3
PCC	PCC(L/R)																											2
SPC	SPC(L/R)																											2
SPD	SPD(L/R)																											3
SPV	SPV(L/R)																											7

This table summarizes the imaging data from Fig. 1. Sites of expression were identified by crossing homeobox reporter alleles into the NeuroPAL (*otIs669* or *otIs696*) landmark strain. Pan-neuronally expressed *ceh-44* and *ceh-48* are not shown here. Numbers on the right show how many homeodomain proteins are expressed in a given neuron class.

**Table 2.** Summary of molecular markers for male-specific neurons

Cardinal neuron class	Class member	*flp-5*	*flp-3*	*flp-7*	*flp-20*	*flp-23*	*flp-27*	*flp-32*	*nlp-1*	*nlp-2*	*nlp-18*	*nlp-50*	*nlp-51*	*ins-2*	*ins-3*	*ins-5*	*ins-6*	*ins-18*	*unc-6*
MCM	MCM(L/R)																		
CEM	CEM(D/V/L/R)																		
CA	CA1																		
	CA2																		
	CA3																		
	CA4																		
	CA5																		
	CA6																		
	CA7																		
	CA8																		
	CA9																		
CP	CP0																		
	CP1																		
	CP2																		
	CP3																		
	CP4																		
	CP5																		
	CP6																		
	CP7																		
	CP8																		
	CP9																		
HOA	HOA																		
HOB	HOB																		
PDC	PDC																		
PGA	PGA																		
PVV	PVV																		
PVY	PVY																		
PVX	PVX																		
PVZ	PVZ																		
DVE	DVE																		
DVF	DVF																		
DX	DX1/2																		
	DX3/4																		
EF	EF1/2																		
	EF3/4																		
RnA	R1A(L/R)																		
	R2A(L/R)																		
	R3A(L/R)																		
	R4A(L/R)																		
	R5A(L/R)																		
	R6A(L/R)																		
	R7A(L/R)																		
	R8A(L/R)																		
	R9A(L/R)																		
RnB	R1B(L/R)																		
	R2B(L/R)																		
	R3B(L/R)																		
	R4B(L/R)																		
	R5B(L/R)																		
	R6B(L/R)																		
	R7B(L/R)																		
	R8B(L/R)																		
	R9B(L/R)																		
PHD	PHD(L/R)																		
PCA	PCA(L/R)																		
PCB	PCB(L/R)																		
PCC	PCC(L/R)																		
SPC	SPC(L/R)																		
SPD	SPD(L/R)																		
SPV	SPV(L/R)																		

This table summarizes the imaging data from Fig. 4. Sites of expression were identified by crossing homeobox reporter alleles into the NeuroPAL (*otIs669* or *otIs696*) landmark strain.

## Data Availability

All relevant data and details of resources can be found within the article and its supplementary information.

## References

[R1] Alexander T, Nolte C and Krumlauf R (2009). Hox genes and segmentation of the hindbrain and axial skeleton. Annu. Rev. Cell Dev. Biol. 25, 431–456. doi:10.1146/annurev.cellbio.042308.113423 19575673

[R2] Berghoff EG, Glenwinkel L, Bhattacharya A, Sun H, Varol E, Mohammadi N, Antone A, Feng Y, Nguyen K, Cook SJ (2021). The Prop1-like homeobox gene unc-42 specifies the identity of synaptically connected neurons. eLife 10, e64903. doi:10.7554/eLife.64903.sa2 34165428 PMC8225392

[R3] Brunschwig K, Wittmann C, Schnabel R, Bürglin TR, Tobler H and Müller F (1999). Anterior organization of the Caenorhabditis elegans embryo by the labial-like Hox gene ceh-13. Development 126, 1537–1546. doi:10.1242/dev.126.7.1537 10068646

[R4] Bürglin TR and Ruvkun G (1993). The Caenorhabditis elegans homeobox gene cluster. Curr. Opin. Genet. Dev. 3, 615–620. doi:10.1016/0959-437X(93)90097-9 7902148

[R5] Chisholm A (1991). Control of cell fate in the tail region of C. elegans by the gene egl-5. Development 111, 921–932. doi:10.1242/dev.111.4.921 1879361

[R6] Chisholm AD, Hutter H, Jin Y and Wadsworth WG (2016). The genetics of axon guidance and axon regeneration in Caenorhabditis elegans. Genetics 204, 849–882. doi:10.1534/genetics.115.186262 28114100 PMC5105865

[R7] Cinar H, Keles S and Jin Y (2005). Expression profiling of GABAergic motor neurons in Caenorhabditis elegans. Curr. Biol. 15, 340–346. doi:10.1016/j.cub.2005.02.025 15723795

[R8] Clark SG, Chisholm AD and Horvitz HR (1993). Control of cell fates in the central body region of C. elegans by the homeobox gene lin-39. Cell 74, 43–55. doi:10.1016/0092-8674(93)90293-Y 8101475

[R9] Cook SJ, Jarrell TA, Brittin CA, Wang Y, Bloniarz AE, Yakovlev MA, Nguyen KCQ, Tang LT-H, Bayer EA, Duerr JS (2019). Whole-animal connectomes of both Caenorhabditis elegans sexes. Nature 571, 63–71. doi:10.1038/s41586-019-1352-7 31270481 PMC6889226

[R10] DeCasien AR, Guma E, Liu S and Raznahan A (2022). Sex differences in the human brain: a roadmap for more careful analysis and interpretation of a biological reality. Biol. Sex. Differ. 13, 43. doi:10.1186/s13293-022-00448-w 35883159 PMC9327177

[R11] Emmons SW (2014). The development of sexual dimorphism: studies of the Caenorhabditis elegans male. Wiley Interdiscipl. Rev. Dev. Biol. 3, 239–262. doi:10.1002/wdev.136 PMC4181595 25262817

[R12] Emmons SW (2018). Neural circuits of sexual behavior in Caenorhabditis elegans. Annu. Rev. Neurosci. 41, 349–369. doi:10.1146/annurev-neuro-070815-014056 29709211

[R13] Feng W, Destain H, Smith JJ and Kratsios P (2022). Maintenance of neurotransmitter identity by Hox proteins through a homeostatic mechanism. Nat. Commun. 13, 6097. doi:10.1038/s41467-022-33781-0 36243871 PMC9569373

[R14] Ferreira HB, Zhang Y, Zhao C and Emmons SW (1999). Patterning of Caenorhabditis elegans posterior structures by the Abdominal-B homolog, egl-5. Dev. Biol. 207, 215–228. doi:10.1006/dbio.1998.9124 10049576

[R15] Garcia LR and Portman DS (2016). Neural circuits for sexually dimorphic and sexually divergent behaviors in Caenorhabditis elegans. Curr. Opin. Neurobiol. 38, 46–52. doi:10.1016/j.conb.2016.02.002 26929998 PMC4921283

[R16] Gendrel M, Atlas EG and Hobert O (2016). A cellular and regulatory map of the GABAergic nervous system of C. elegans. eLife 5, e17686. doi:10.7554/eLife.17686 27740909 PMC5065314

[R17] Haque R, Setty H, Lorenzo R, Stelzer G, Rotkopf R, Salzberg Y, Goldman G, Kumar S, Halber SN, Leifer AM (2025). Decoding sexual dimorphism of the sex-shared nervous system at single-neuron resolution. Sci. Adv. 11, eadv9106. doi:10.1126/sciadv.adv9106 40644535 PMC12248294

[R18] Hobert O (2016). Terminal selectors of neuronal identity. Curr. Top. Dev. Biol. 116, 455–475. doi:10.1016/bs.ctdb.2015.12.007 26970634

[R19] Hobert O (2021). Homeobox genes and the specification of neuronal identity. Nat. Rev. Neurosci. 22, 627–636. doi:10.1038/s41583-021-00497-x 34446866

[R20] Hobert O, Tessmar K and Ruvkun G (1999). The Caenorhabditis elegans lim-6 LIM homeobox gene regulates neurite outgrowth and function of particular GABAergic neurons. Development 126, 1547–1562. doi:10.1242/dev.126.7.1547 10068647

[R21] Hodgkin J (1983). Male phenotypes and mating efficiency in Caenorhabditis elegans. Genetics 103, 43–64. doi:10.1093/genetics/103.1.43 17246100 PMC1202023

[R22] Howell K and Hobert O (2017). Morphological diversity of C. elegans sensory cilia instructed by the differential expression of an immunoglobulin domain protein. Curr. Biol. 27, 1782–1790.e1785. doi:10.1016/j.cub.2017.05.006 28578929

[R23] Hueber SD, Weiller GF, Djordjevic MA and Frickey T (2010). Improving Hox protein classification across the major model organisms. PLoS ONE 5, e10820. doi:10.1371/journal.pone.0010820 20520839 PMC2876039

[R24] Iosilevskii Y, Hall DH, Katz M and Podbilewicz B (2025). The PVD neuron has male-specific structure and mating function in Caenorhabditis elegans. Proc. Natl. Acad. Sci. USA 122, e2421376122. doi:10.1073/pnas.2421376122 40138342 PMC12002248

[R25] Jarrell TA, Wang Y, Bloniarz AE, Brittin CA, Xu M, Thomson JN, Albertson DG, Hall DH and Emmons SW (2012). The connectome of a decision-making neural network. Science 337, 437–444. doi:10.1126/science.1221762 22837521

[R26] Jin Y, Hoskins R and Horvitz HR (1994). Control of type-D GABAergic neuron differentiation by C. elegans UNC-30 homeodomain protein. Nature 372, 780–783. doi:10.1038/372780a0 7997265

[R27] Kalis AK, Kissiov DU, Kolenbrander ES, Palchick Z, Raghavan S, Tetreault BJ, Williams E, Loer CM and Wolff JR (2014). Patterning of sexually dimorphic neurogenesis in the caenorhabditis elegans ventral cord by Hox and TALE homeodomain transcription factors. Dev. Dyn. 243, 159–171. doi:10.1002/dvdy.24064 24115648

[R28] Kalis AK, Sterrett MC, Armstrong C, Ballmer A, Burkstrand K, Chilson E, Emlen E, Ferrer E, Loeb S, Olin T (2022). Hox proteins interact to pattern neuronal subtypes in Caenorhabditis elegans males. Genetics 220, iyac010. doi:10.1093/genetics/iyac010 35137058 PMC8982040

[R29] Kim K and Li C (2004). Expression and regulation of an FMRFamide-related neuropeptide gene family in Caenorhabditis elegans. J. Comp. Neurol. 475, 540–550. doi:10.1002/cne.20189 15236235

[R30] Kirangwa J, Laetsch DR, King E, Stevens L, Blaxter M, Holovachov O and Schiffer P (2024). Evolutionary plasticity in nematode Hox gene complements and genomic loci arrangement. Sci. Rep. 14, 29513. doi:10.1038/s41598-024-79962-3 39604390 PMC11603191

[R31] Kratsios P, Stolfi A, Levine M and Hobert O (2011). Coordinated regulation of cholinergic motor neuron traits through a conserved terminal selector gene. Nat. Neurosci. 15, 205–214. doi:10.1038/nn.2989 22119902 PMC3267877

[R32] Kratsios P, Kerk SY, Catela C, Liang J, Vidal B, Bayer EA, Feng W, De La Cruz ED, Croci L, Consalez GG (2017). An intersectional gene regulatory strategy defines subclass diversity of C. elegans motor neurons. eLife 6, e25751. doi:10.7554/eLife.25751 28677525 PMC5498135

[R33] Krumlauf R (2018). Hox genes, clusters and collinearity. Int. J. Dev. Biol. 62, 659–663. doi:10.1387/ijdb.180330rr 30604835

[R34] Leyva-Diaz E and Hobert O (2022). Robust regulatory architecture of pan-neuronal gene expression. Curr. Biol. 32, 1715–1727. doi:10.1016/j.cub.2022.02.040 35259341 PMC9050922

[R35] Li H, Janssens J, De Waegeneer M, Kolluru SS, Davie K, Gardeux V, Saelens W, David FPA, Brbić M, Spanier K (2022). Fly Cell Atlas: a single-nucleus transcriptomic atlas of the adult fruit fly. Science 375, eabk2432. doi:10.1126/science.abk2432 35239393 PMC8944923

[R36] Lints R and Emmons SW (1999). Patterning of dopaminergic neurotransmitter identity among Caenorhabditis elegans ray sensory neurons by a TGFbeta family signaling pathway and a Hox gene. Development 126, 5819–5831. doi:10.1242/dev.126.24.5819 10572056

[R37] Lints R and Emmons SW (2002). Regulation of sex-specific differentiation and mating behavior in C. elegans by a new member of the DM domain transcription factor family. Genes Dev. 16, 2390–2402. doi:10.1101/gad.1012602 12231628 PMC187445

[R38] Lints R, Jia L, Kim K, Li C and Emmons SW (2004). Axial patterning of C. elegans male sensilla identities by selector genes. Dev. Biol. 269, 137–151. doi:10.1016/j.ydbio.2004.01.021 15081363

[R39] Liu KS and Sternberg PW (1995). Sensory regulation of male mating behavior in Caenorhabditis elegans. Neuron 14, 79–89. doi:10.1016/0896-6273(95)90242-2 7826644

[R40] Loer CM and Kenyon CJ (1993). Serotonin-deficient mutants and male mating behavior in the nematode Caenorhabditis elegans. J. Neurosci. 13, 5407–5417. doi:10.1523/JNEUROSCI.13-12-05407.1993 8254383 PMC6576401

[R41] Ma X, Zhao Z, Xiao L, Xu W, Kou Y, Zhang Y, Wu G, Wang Y and Du Z (2021). A 4D single-cell protein atlas of transcription factors delineates spatiotemporal patterning during embryogenesis. Nat. Methods 18, 893–902. doi:10.1038/s41592-021-01216-1 34312566

[R42] Merabet S and Galliot B (2015). The TALE face of Hox proteins in animal evolution. Front. Genet. 6, 267. doi:10.3389/fgene.2015.00267 26347770 PMC4539518

[R43] Moens CB and Selleri L (2006). Hox cofactors in vertebrate development. Dev. Biol. 291, 193–206. doi:10.1016/j.ydbio.2005.10.032 16515781

[R44] Molina-Garcia L, Lloret-Fernández C, Cook SJ, Kim B, Bonnington RC, Sammut M, O’Shea JM, Gilbert SP, Elliott DJ, Hall DH (2020). Direct glia-to-neuron transdifferentiation gives rise to a pair of male-specific neurons that ensure nimble male mating. eLife 9, e48631. doi:10.7554/eLife.48361 PMC7609048 33138916

[R45] Morillo KS, St Ange J, Weng Y, Kaletsky R and Murphy CT (2025). Single-nucleus neuronal transcriptional profiling of male C. elegans uncovers regulators of sex-specific and sex-shared behaviors. Cell Rep. 44, 116016. doi:10.1016/j.celrep.2025.116016 40682776 PMC12908727

[R46] Nehme R, Grote P, Tomasi T, Loser S, Holzkamp H, Schnabel R and Conradt B (2010). Transcriptional upregulation of both egl-1 BH3-only and ced-3 caspase is required for the death of the male-specific CEM neurons. Cell Death Differ. 17, 1266–1276. doi:10.1038/cdd.2010.3 20150917 PMC2902690

[R47] Peden E, Kimberly E, Gengyo-Ando K, Mitani S and Xue D (2007). Control of sex-specific apoptosis in C. elegans by the BarH homeodomain protein CEH-30 and the transcriptional repressor UNC-37/Groucho. Genes Dev. 21, 3195–3207. doi:10.1101/gad.1607807 18056429 PMC2081983

[R48] Pereira L, Kratsios P, Serrano-Saiz E, Sheftel H, Mayo AE, Hall DH, White JG, LeBoeuf B, Garcia LR, Alon U (2015). A cellular and regulatory map of the cholinergic nervous system of C. elegans. eLife 4, 12342. doi:10.7554/eLife.12432 PMC4769160 26705699

[R49] Philippidou P and Dasen JS (2013). Hox genes: choreographers in neural development, architects of circuit organization. Neuron 80, 12–34. doi:10.1016/j.neuron.2013.09.020 24094100 PMC3835187

[R50] Reilly MB, Cros C, Varol E, Yemini E and Hobert O (2020). Unique homeobox codes delineate all the neuron classes of C. elegans. Nature 584, 595–601. doi:10.1038/s41586-020-2618-9 32814896 PMC7587405

[R51] Reilly MB, Tekieli T, Cros C, Aguilar GR, Lao J, Toker IA, Vidal B, Leyva-Diaz E, Bhattacharya A, Cook SJ (2022). Widespread employment of conserved C. elegans homeobox genes in neuronal identity specification. PLoS Genet. 18, e1010372. doi:10.1371/journal.pgen.1010372 36178933 PMC9524666

[R52] Ripoll-Sanchez L, Watteyne J, Sun H, Fernandez R, Taylor SR, Weinreb A, Bentley BL, Hammarlund M, Miller DM III, Hobert O (2023). The neuropeptidergic connectome of C. elegans. Neuron 111, 3570–3589.e3575. doi:10.1016/j.neuron.2023.09.043 37935195 PMC7615469

[R53] Salser SJ, Loer CM and Kenyon C (1993). Multiple HOM-C gene interactions specify cell fates in the nematode central nervous system. Genes Dev. 7, 1714–1724. doi:10.1101/gad.7.9.1714 8103754

[R54] Sammut M, Cook SJ, Nguyen KCQ, Felton T, Hall DH, Emmons SW, Poole RJ and Barrios A (2015). Glia-derived neurons are required for sex-specific learning in C. elegans. Nature 526, 385–390. doi:10.1038/nature15700 26469050 PMC4650210

[R55] Schindelin J, Arganda-Carreras I, Frise E, Kaynig V, Longair M, Pietzsch T, Preibisch S, Rueden C, Saalfeld S, Schmid B (2012). Fiji: an open-source platform for biological-image analysis. Nat. Methods 9, 676–682. doi:10.1038/nmeth.2019 22743772 PMC3855844

[R56] Schwartz HT and Horvitz HR (2007). The C. elegans protein CEH-30 protects male-specific neurons from apoptosis independently of the Bcl-2 homolog CED-9. Genes Dev. 21, 3181–3194. doi:10.1101/gad.1607007 18056428 PMC2081982

[R57] Serrano-Saiz E, Gulez B, Pereira L, Gendrel M, Kerk SY, Vidal B, Feng W, Wang C, Kratsios P, Rand JB (2020). Modular organization of cis-regulatory control information of neurotransmitter pathway genes in Caenorhabditis elegans. Genetics 215, 665–681. doi:10.1534/genetics.120.303206 32444379 PMC7337081

[R58] Shaham S and Bargmann CI (2002). Control of neuronal subtype identity by the C. elegans ARID protein CFI-1. Genes Dev. 16, 972–983. doi:10.1101/gad.976002 11959845 PMC152356

[R59] Sherlekar AL, Janssen A, Siehr MS, Koo PK, Caflisch L, Boggess M and Lints R (2013). The C. elegans male exercises directional control during mating through cholinergic regulation of sex-shared command interneurons. PLoS ONE 8, e60597. doi:10.1371/journal.pone.0060597 23577128 PMC3618225

[R60] Siehr MS, Koo PK, Sherlekar AL, Bian X, Bunkers MR, Miller RM, Portman DS and Lints R (2011). Multiple doublesex-related genes specify critical cell fates in a C. elegans male neural circuit. PLoS ONE 6, e26811. doi:10.1371/journal.pone.0026811 22069471 PMC3206049

[R61] Smith JJ and Kratsios P (2024). Hox gene functions in the C. elegans nervous system: From early patterning to maintenance of neuronal identity. Semin. Cell Dev. Biol. 152-153, 58–69. doi:10.1016/j.semcdb.2022.11.012 36496326 PMC10244487

[R62] Smith JJ, Taylor SR, Blum JA, Feng W, Collings R, Gitler AD, Miller DM III and Kratsios P (2024). A molecular atlas of adult C. elegans motor neurons reveals ancient diversity delineated by conserved transcription factor codes. Cell Rep. 43, 113857. doi:10.1016/j.celrep.2024.113857 38421866 PMC11091551

[R63] Sulston JE, Albertson DG and Thomson JN (1980). The Caenorhabditis elegans male: postembryonic development of nongonadal structures. Dev. Biol. 78, 542–576. doi:10.1016/0012-1606(80)90352-8 7409314

[R64] Sulston JE, Schierenberg E, White JG and Thomson JN (1983). The embryonic cell lineage of the nematode Caenorhabditis elegans. Dev. Biol. 100, 64–119. doi:10.1016/0012-1606(83)90201-4 6684600

[R65] Taylor SR, Santpere G, Weinreb A, Barrett A, Reilly MB, Xu C, Varol E, Oikonomou P, Glenwinkel L, McWhirter R (2021). Molecular topography of an entire nervous system. Cell 184, 4329–4347.e4323. doi:10.1016/j.cell.2021.06.023 34237253 PMC8710130

[R66] Tekieli T, Yemini E, Nejatbakhsh A, Wang C, Varol E, Fernandez RW, Masoudi N, Paninski L and Hobert O (2021). Visualizing the organization and differentiation of the male-specific nervous system of C. elegans. Development 148, dev199687. doi:10.1242/dev.199687 34415309 PMC8489020

[R67] Wang C, Vidal B, Sural S, Loer C, Aguilar GR, Merritt DM, Toker IA, Vogt MC, Cros CC and Hobert O (2024). A neurotransmitter atlas of C. elegans males and hermaphrodites. eLife 13, RP95402. doi:10.7554/eLife.95402.3 39422452 PMC11488851

[R68] Wittmann C, Bossinger O, Goldstein B, Fleischmann M, Kohler R, Brunschwig K, Tobler H and Müller F (1997). The expression of the C. elegans labial-like Hox gene ceh-13 during early embryogenesis relies on cell fate and on anteroposterior cell polarity. Development 124, 4193–4200. doi:10.1242/dev.124.21.4193 9334268

[R69] Yang Y, Sun Y, Luo X, Zhang Y, Chen Y, Tian E, Lints R and Zhang H (2007). Polycomb-like genes are necessary for specification of dopaminergic and serotonergic neurons in Caenorhabditis elegans. Proc. Natl. Acad. Sci. USA 104, 852–857. doi:10.1073/pnas.0610261104 17215367 PMC1783403

[R70] Yao Z, van Velthoven CTJ, Kunst M, Zhang M, McMillen D, Lee C, Jung W, Goldy J, Abdelhak A, Aitken M (2023). A high-resolution transcriptomic and spatial atlas of cell types in the whole mouse brain. Nature 624, 317–332. doi:10.1038/s41586-023-06812-z 38092916 PMC10719114

[R71] Yemini E, Lin A, Nejatbakhsh A, Varol E, Sun R, Mena GE, Samuel ADT, Paninski L, Venkatachalam V and Hobert O (2021). NeuroPAL: a multicolor atlas for whole-brain neuronal identification in C. elegans. Cell 184, 272–288.e211. doi:10.1016/j.cell.2020.12.012 33378642 PMC10494711

[R72] Yu H, Pretot RF, Burglin TR and Sternberg PW (2003). Distinct roles of transcription factors EGL-46 and DAF-19 in specifying the functionality of a polycystin-expressing sensory neuron necessary for C. elegans male vulva location behavior. Development 130, 5217–5227. doi:10.1242/dev.00678 12954713

[R73] Yu H, Seah A, Herman MA, Ferguson EL, Horvitz HR and Sternberg PW (2009). Wnt and EGF pathways act together to induce C. elegans male hook development. Dev. Biol. 327, 419–432. doi:10.1016/j.ydbio.2008.12.023 19154732 PMC2695933

