# Supplementary material for: Pervasive homeobox gene function in the male-specific nervous system of Caenorhabditis elegans
Source: Development. Author manuscript; Available in PMC 2026 Feb 28. (PMC12949664; doi:10.1242/dev.204958)
Supplement: All Supp Figs and Tables [file NIHMS2137783-supplement-All_Supp_Figs_and_Tables.pdf]

SUPPLEMENTARY FIGURES AND TABLES

Fig. S1: NeuroPAL ID data for homeobox reporter alleles

Homeobox reporter alleles from Fig. 1 merged with NeuroPAL colors. Panels show representative images of NeuroPAL (*otIs669* or *otIs696*) landmark allele where all pseudocolors, as described (Tekieli et al., 2021; Yemini et al., 2021), are merged together with the GFP signal of homeobox reporters. Circles indicate the nuclear signal, whilst arrows are used to match the signal to the specific Neural ID. White dotted lines are used to trace the contours of the animal and its pharynx. Multiple images acquired with the NeuroPAL landmark are used to obtain a neuronal ID that is overlaid on representative images.

Fig. S2: NeuroPAL ID data for HOX cluster alleles

HOX cluster reporter alleles from Fig. 2 merged with NeuroPAL colors. Panels show representative images of NeuroPAL (*otIs669* or *otIs696*) landmark allele where all pseudocolors, as described (Tekieli et al., 2021; Yemini et al., 2021), are merged together with the GFP signal of HOX reporters. Circles indicate the nuclear signal, whilst arrows are used to match the signal to the specific Neural ID. White dotted lines are used to trace the contours of the animal and its pharynx. Multiple images acquired with the NeuroPAL landmark are used to obtain a neuronal ID that is overlaid on representative images.

Fig. S3: NeuroPAL ID data for new terminal identity markers for male-specific neurons

A,B: New terminal identity markers for male-specific neurons shown in Fig. 4 merged with NeuroPAL colors. Panels show representative images of NeuroPAL (*otIs669* or *otIs696*) landmark allele where all pseudocolors, as described (Tekieli et al., 2021; Yemini et al., 2021), are merged together. Circles indicate the nuclear signal, whilst arrows are used to match the signal to the specific Neural ID. White dotted lines are used to trace the contours of the animal and its pharynx. Multiple images acquired with

the NeuroPAL landmark are used to obtain a neuronal ID that is overlaid on representative images.

Fig. S4: *ttx-1* and *lin-11* mutant analysis

A: *lin-11* full deletion mutants affect the expression *of unc-6/Netrin* in PHD. Representative images show the expression of *unc-6(syb5064)*, *nlp-51(syb3936)*, NeuroPAL colors(*otIs669*) and a merge of both in wild type and *lin-11(ot1448)* null mutants. Bar graph quantifies the decrease in Relative Intensity Value of *unc-6(*syb5064) and *nlp-51(syb3936)* expression in PHD and PVX, PHD respectively.

B: *ttx-1 cis*-regulatory mutants affect the expression of *unc-6/Netrin* in PHD. Representative images show the expression of *unc-6(syb5064))*, NeuroPAL colors(*otIs669*) and a merge of both in wild type and *ttx-1(syb1679 ot1264)* mutants. Bar graph quantifies the decrease in Relative Intensity Value of *unc-6(syb5064)* expression in PHD.

Statistics: *p value ≤0.05, ***p value ≤0.001, ****p value ≤0.0001, ns, not significant. Testing was performed using the Student t- test.

Table S1: List of all *C. elegans* homeodomain proteins. The table indicates which of the 102 *C. elegans* homeodomain proteins were analyzed for expression in the male-specific nervous system.

Table S2: Strain list. List of all strains used in the manuscript.

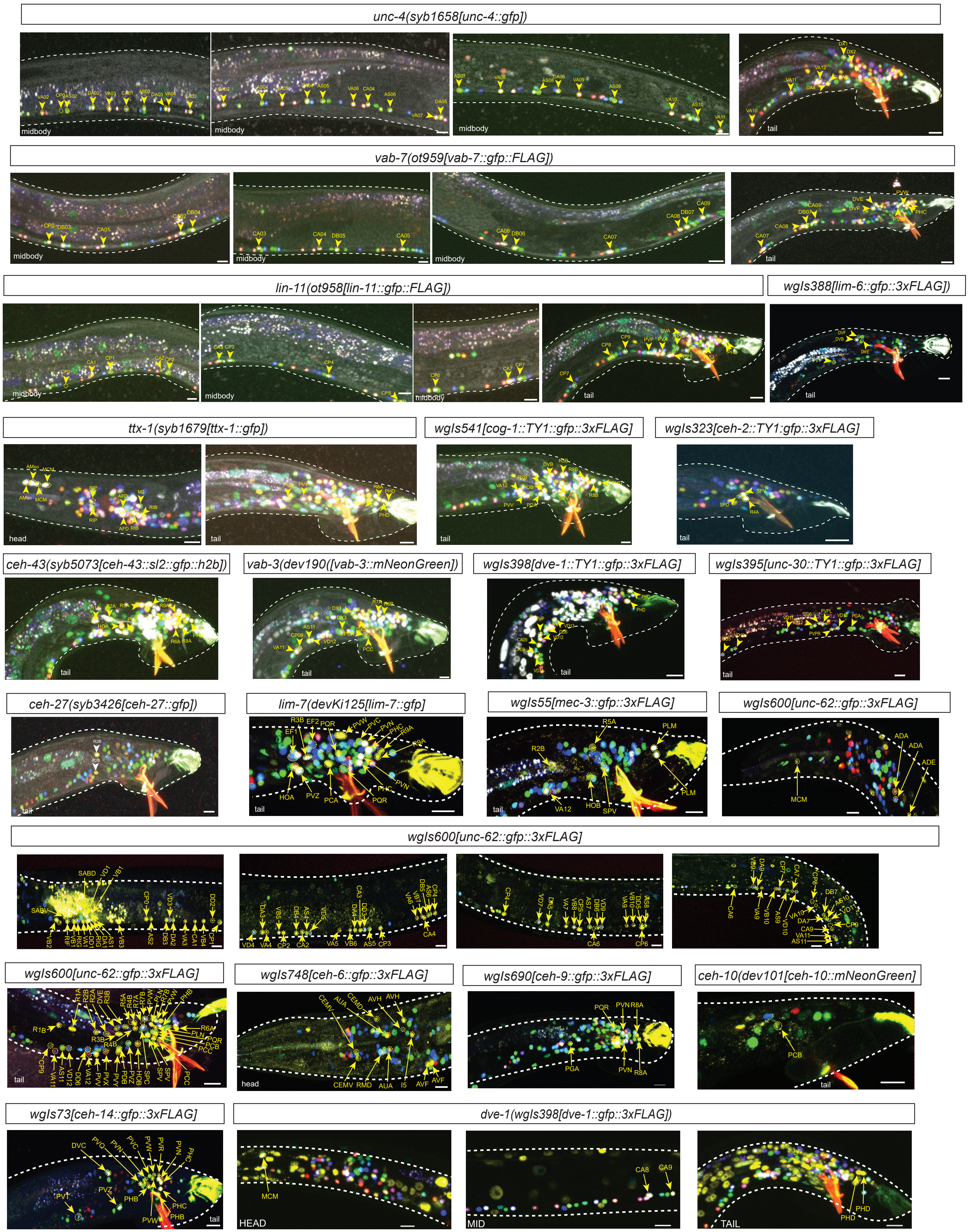
unc-4(syb1658[unc-4::gfp])
vab-7(ot959[vab-7::gfp::FLAG])
lin-11(ot958[lin-11::gfp::FLAG])
wgIs388[lim-6::gfp::3xFLAG])
ttx-1(syb1679[ttx-1::gfp])
wgIs541[cog-1::TY1::gfp::3xFLAG]
wgIs323[ceh-2::TY1:gfp::3xFLAG]
ceh-43(syb5073[ceh-43::sl2::gfp::h2b])
vab-3(dev190([vab-3::mNeonGreen])
wgIs398[dve-1::TY1::gfp::3xFLAG]
wgIs395[unc-30::TY1::gfp::3xFLAG]
ceh-27(syb3426[ceh-27::gfp])
lim-7(devKi125[lim-7::gfp]
wgIs55[mec-3::gfp::3xFLAG]
wgIs600[unc-62::gfp::3xFLAG]
wgIs748[ceh-6::gfp::3xFLAG]
wgIs690[ceh-9::gfp::3xFLAG]
ceh-10(dev101[ceh-10::mNeonGreen]
wgIs73[ceh-14::gfp::3xFLAG]
dve-1(wgIs398[dve-1::gfp::3xFLAG])
midbody
tail
head
HEAD
MID
TAIL

Fig.S1

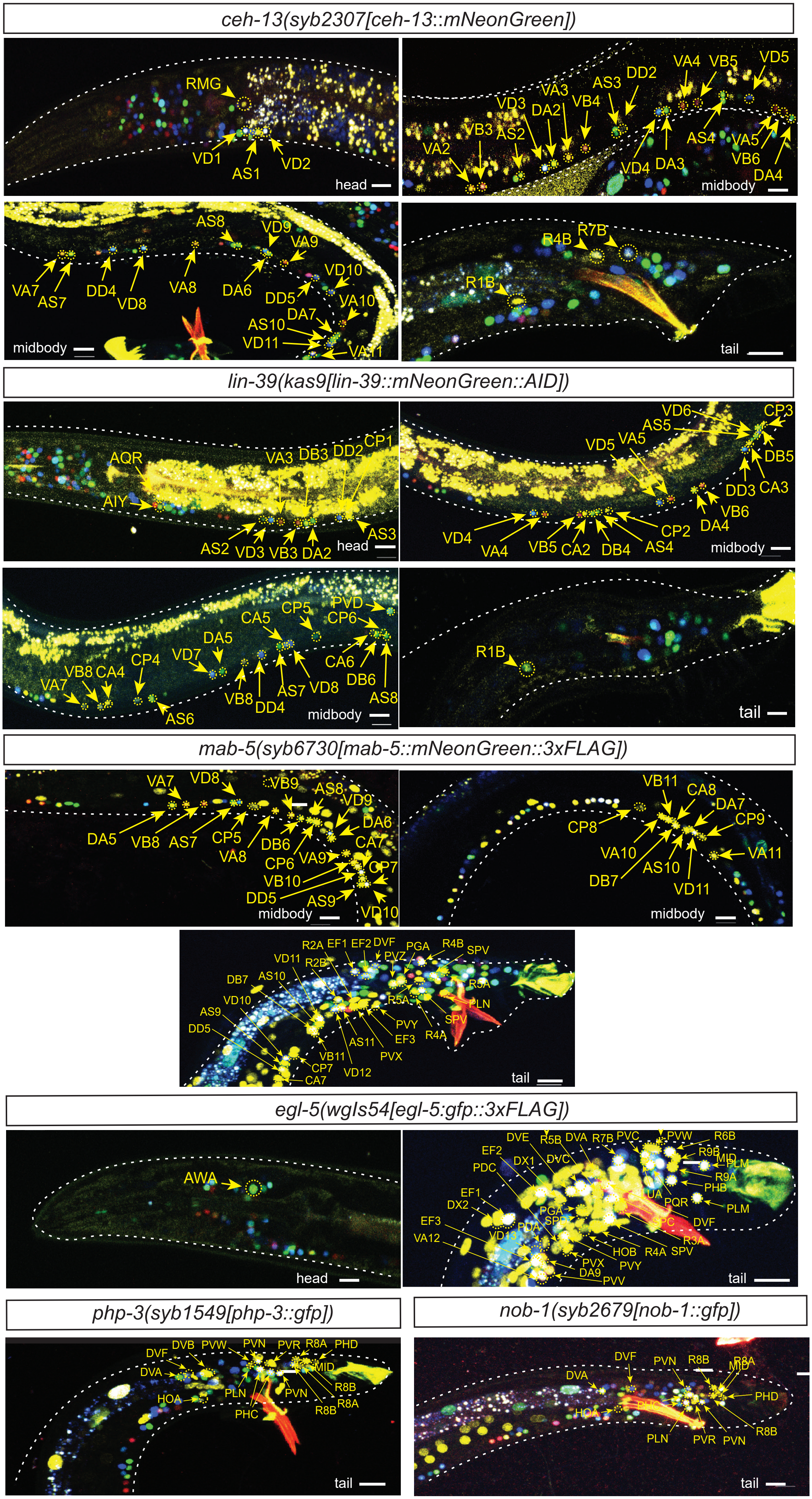

ceh-13(syb2307[ceh-13::mNeonGreen])
head
midbody
midbody
tail
lin-39(kas9[lin-39::mNeonGreen::AID])
head
midbody
midbody
tail
mab-5(syb6730[mab-5::mNeonGreen::3xFLAG])
midbody
midbody
tail
egl-5(wgIs54[egl-5:gfp::3xFLAG])
head
tail
php-3(syb1549[php-3::gfp])
tail
nob-1(syb2679[nob-1::gfp])
tail

Fig.S2

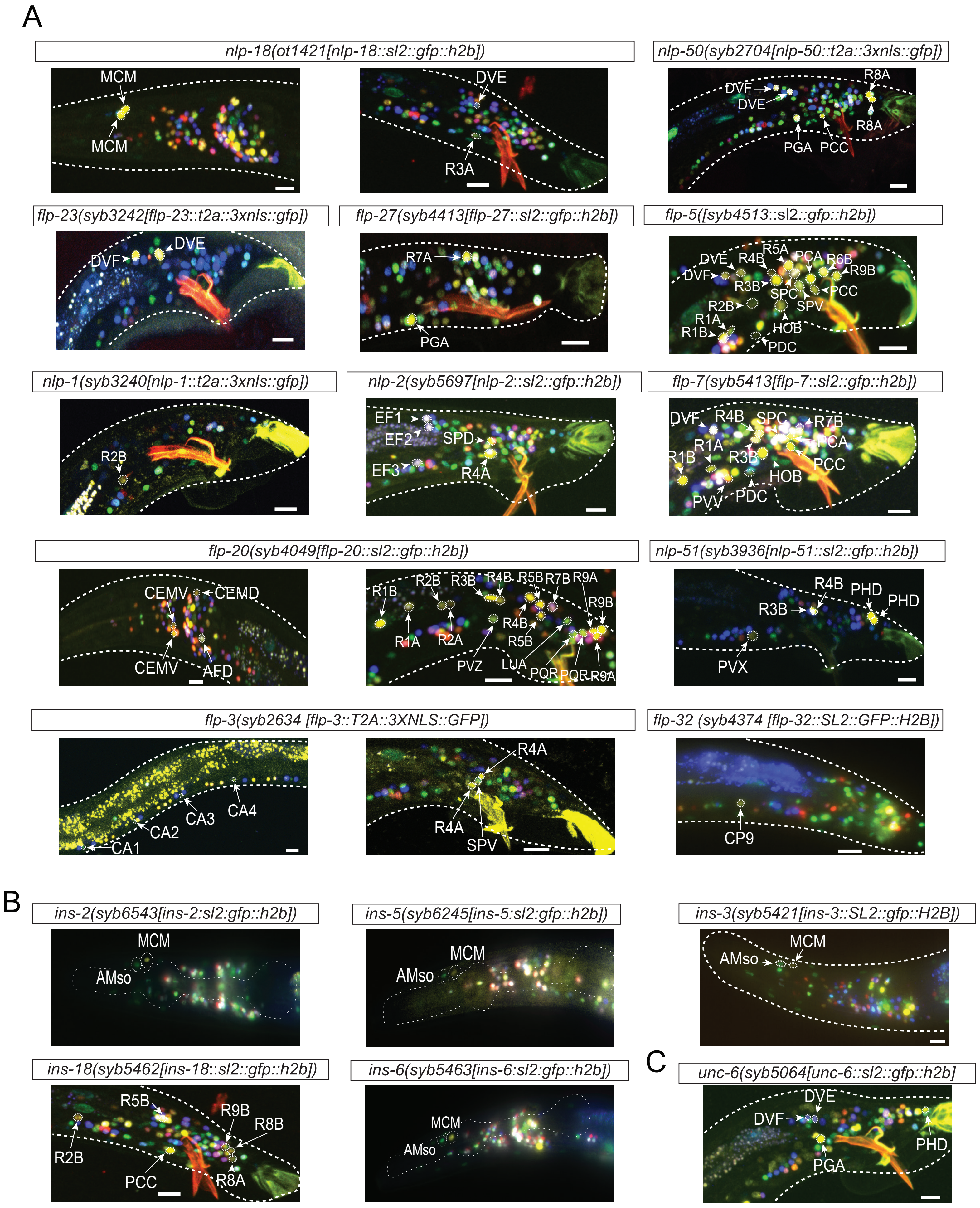
A
nlp-18(ot1421[nlp-18::sl2::gfp::h2b])
MCM
MCM
DVE
R3A
nlp-50(syb2704[nlp-50::t2a::3xnls::gfp])
DVF
DVE
R8A
R8A
PGA
PCC
flp-23(syb3242[flp-23::t2a::3xnls::gfp])
DVF
DVE
flp-27(syb4413[flp-27::sl2::gfp::h2b])
R7A
PGA
flp-5([syb4513::sl2::gfp::h2b])
DVE
R4B
R5A
PCA
R6B
R9B
DVF
R3B
SPC
PCC
SPV
R2B
R1A
R1B
HOB
PDC
nlp-1(syb3240[nlp-1::t2a::3xnls::gfp])
R2B
nlp-2(syb5697[nlp-2::sl2::gfp::h2b])
EF1
EF2
SPD
EF3
R4A
flp-7(syb5413[flp-7::sl2::gfp::h2b])
DVF
R4B
SPC
R7B
R1A
PCA
R1B
R3B
PCC
HOB
PVV
PDC
flp-20(syb4049[flp-20::sl2::gfp::h2b])
CEMV
CEMD
CEMV
AFD
R1B
R2B
R3B
R4B
R5B
R7B
R9A
R9B
R1A
R2A
R4B
R5B
PVZ
LUA
PQR
PQR
R9A
nlp-51(syb3936[nlp-51::sl2::gfp::h2b])
R4B
PHD
PHD
R3B
PVX
flp-3(syb2634 [flp-3::T2A::3XNLS::GFP])
CA4
CA3
CA2
CA1
R4A
R4A
SPV
flp-32 (syb4374 [flp-32::SL2::GFP::H2B])
CP9
B
ins-2(syb6543[ins-2:sl2:gfp::h2b])
MCM
AMso
ins-5(syb6245[ins-5:sl2:gfp::h2b])
MCM
AMso
ins-3(syb5421[ins-3::SL2::gfp::H2B])
MCM
AMso
ins-18(syb5462[ins-18::sl2::gfp::h2b])
R5B
R9B
R8B
R2B
PCC
R8A
ins-6(syb5463[ins-6:sl2:gfp::h2b])
MCM
AMso
C
unc-6(syb5064[unc-6::sl2::gfp::h2b]
DVE
DVF
PHD
PGA

Fig.S3

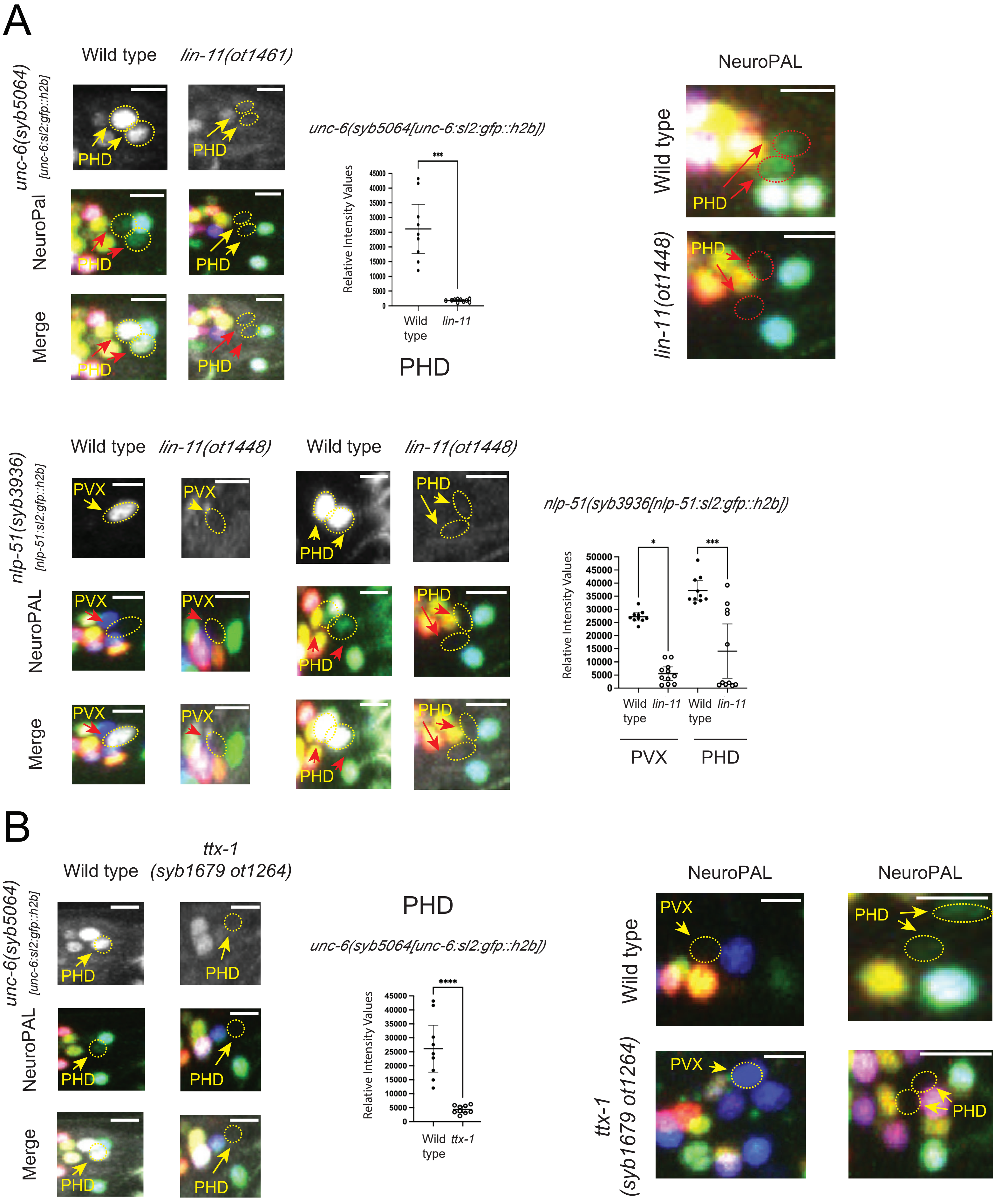
A
Wild type
lin-11(ot1461)
unc-6(syb5064) [unc-6:sl2:gfp::h2b]
NeuroPal
Merge
PHD
unc-6(syb5064[unc-6:sl2:gfp::h2b])
Relative Intensity Values
***
Wild type
lin-11
PHD
NeuroPAL
Wild type
lin-11(ot1448)
Wild type
lin-11(ot1448)
Wild type
lin-11(ot1448)
nlp-51(syb3936) [nlp-51:sl2:gfp::h2b]
NeuroPAL
Merge
PVX
PHD
nlp-51(syb3936[nlp-51:sl2:gfp::h2b])
Relative Intensity Values
*
***
Wild type
lin-11
Wild type
lin-11
PVX
PHD
B
Wild type
ttx-1 (syb1679 ot1264)
unc-6(syb5064) [unc-6:sl2:gfp::h2b]
NeuroPAL
Merge
PHD
unc-6(syb5064[unc-6:sl2:gfp::h2b])
Relative Intensity Values
****
Wild type
ttx-1
NeuroPAL
NeuroPAL
Wild type
ttx-1 (syb1679 ot1264)
PVX
PHD

Fig.S4

Table S1: List of all C. elegans homeobox genes and information about expression analysis

Subclass	Gene	Strain	Reporter	Examined
**CONSERVED**				
ANTP	ceh-12	SYS629	CRISPR	
ANTP	ceh-16	PHX2280	CRISPR	yes
ANTP	ceh-2	SYS606	CRISPR	yes
ANTP	ceh-23	PHX1849	CRISPR	
ANTP	ceh-43	PHX5073	CRISPR	yes
ANTP	ceh-5	PHX1592	CRISPR	yes
ANTP	ceh-62	OP416	Fosmid	
ANTP	ceh-63	OP742	Fosmid	
ANTP	ceh-7	OP168	Fosmid	
ANTP	pha-2	OP687	Fosmid	
ANTP-HOXL	ceh-13	OH16366	Fosmid	yes
ANTP-HOXL	egl-5	OP54	Fosmid	yes
ANTP-HOXL	lin-39	KRA103	CRISPR	yes
ANTP-HOXL	mab-5	OP27	Fosmid	yes
ANTP-HOXL	nob-1	JIM271	Fosmid	yes
ANTP-HOXL	pal-1	OP380	Fosmid	
ANTP-HOXL	php-3	PHX1549	CRISPR	yes
ANTP-HOXL	vab-7	OH15912	CRISPR	yes
ANTP-NKL	ceh-1	OP571	Fosmid	
ANTP-NKL	ceh-19	OP739	Fosmid	yes
ANTP-NKL	ceh-22	OP389	Fosmid	
ANTP-NKL	ceh-24	PHX1608	CRISPR	yes
ANTP-NKL	ceh-27	PHX3426	CRISPR	yes
ANTP-NKL	ceh-28	SYS635	CRISPR	
ANTP-NKL	ceh-30	PHX4678	CRISPR	
ANTP-NKL	ceh-31	OH17791	CRISPR	
ANTP-NKL	ceh-51	PHX1551	CRISPR	
ANTP-NKL	ceh-9	OP690	Fosmid	yes
ANTP-NKL	cog-1	OP541	Fosmid	yes
ANTP-NKL	mls-2	OP645	Fosmid	yes
ANTP-NKL	tab-1	PHX1587	CRISPR	
ANTP-NKL	vab-15	OP730	Fosmid	
CUT	ceh-21	OP759	Fosmid	
CUT	ceh-38	OP241	Fosmid	
CUT	ceh-39	OP759	Fosmid	
CUT	ceh-41	OP759	Fosmid	
CUT	ceh-44	OH16219	CRISPR	yes
CUT	ceh-48	OP631	Fosmid	yes
CUT	ceh-49	OH16224	CRISPR	
CUT	dve-1	OP398	Fosmid	yes
HNF	hmbx-1	OP655	Fosmid	
LIM	ceh-14	OP73	Fosmid	yes
LIM	lim-4	OP681	Fosmid	yes
LIM	lim-6	OP387	Fosmid	yes
LIM	lim-7	devKi125	CRISPR	yes
LIM	lin-11	OH15910	CRISPR	yes
LIM	mec-3	OP55	Fosmid	yes
LIM	ttx-3	Vincent Bertrand, pers. Comm.	CRISPR	yes
POU	ceh-18	OP533	Fosmid	
POU	ceh-6	RW10871	Fosmid	yes
POU	unc-86		CRISPR	
PRD	eyg-1	OP441	Fosmid	
PRD	pax-3	OP190	Fosmid	
PRD	vab-3	devKi190	CRISPR	yes
PRD-like	alr-1	OP200	Fosmid	
PRD-like	ceh-10	SYS427	CRISPR	yes
PRD-like	ceh-17	SYS608	CRISPR	yes
PRD-like	ceh-36	PHX2934	CRISPR	yes
PRD-like	ceh-37	OH16345	CRISPR	yes
PRD-like	ceh-45	SYS634	CRISPR	
PRD-like	ceh-53	OP444	Fosmid	
PRD-like	ceh-54	OP456	Fosmid	
PRD-like	ceh-8	PHX1656	CRISPR	yes
PRD-like	dsc-1	OP522	Fosmid	
PRD-like	ttx-1	PHX1679	CRISPR	yes
PRD-like	unc-30	SYS743	CRISPR	yes
PRD-like	unc-4	PHX1658	CRISPR	yes
PRD-like	unc-42		CRISPR	yes
PROS	pros-1	OP500	Fosmid	
SO/SIX	ceh-32	OH16477	CRISPR	
SO/SIX	ceh-33	OP575	Fosmid	
SO/SIX	ceh-34	OP524	Fosmid	yes
SO/SIX	unc-39	OP186	Fosmid	
PBC	ceh-20	RW12211	CRISPR	
PBC	ceh-40	OP232	Fosmid	
PBC	ceh-60	DLS395	CRISPR	
IRO	irx-1	OP536	Fosmid	
MEIS	unc-62	SD1871	Fosmid	yes
ZF	zag-1	OP83	Fosmid	
ZF	zfh-2	OH16346	CRISPR	
**NOT CONSERVED**				
Divergent	ceh-100	OH16488	CRISPR	
Divergent	ceh-57	OP706	Fosmid	
Divergent	ceh-58	PHX2015	CRISPR	
Divergent	ceh-74	OP680	Fosmid	
Divergent	ceh-75	PHX1884	CRISPR	
Divergent	ceh-76	OH16487	CRISPR	
Divergent	ceh-79	OP553	Fosmid	
Divergent	ceh-81	OH16479	Fosmid	
Divergent	ceh-82	SYS609	CRISPR	
Divergent	ceh-83	OP727	Fosmid	
Divergent	ceh-84	PHX2811	CRISPR	
Divergent	ceh-86	PHX2517	CRISPR	
Divergent	ceh-87	PHX1955	CRISPR	
Divergent	ceh-88	OP593	Fosmid	
Divergent	ceh-89	OH16505	CRISPR	
Divergent	ceh-90	OP210	Fosmid	
Divergent	ceh-91	OH16480	Fosmid	
Divergent	ceh-92	PHX1610	Fosmid	
Divergent	ceh-93	OP554	Fosmid	
Divergent	ceh-99	OH16481	Fosmid	
Divergent	duxl-1	OP470	Fosmid	
Divergent	nsy-7	OH16371	Fosmid	

examined, expressed in male-specific neurons	28 genes
examined, not expressed in male-specific neurons	12 genes
total examined	40 genes conserved out of 80 conserved total

Table S2 Strain list

Strain	Genotype of mutant or knock-in	Reference
BC12852	dpy-5(e907) I; sEx12852[grl-2p::GFP + pCeh361]	PMID: 15338614
CB4088	him-5(e1490)V	PMID: 17248881
CHL67	lin-48(drpIs3 [lin-48p::tdTomato]) I; oig-1(drpIs4 [oig-8p::GFP + pha-1(+)])III; him-5(e1490)V	PMID: 33138916
LC188	unc-30(ot1186) IV; him-5(e1490) V	This study
MT633	lin-11(n389) I; him-5(e1467)V	PMID: 3996896
OH13589	otIs450 [oig-1 fosmid::SL2::GFP; rol-6(+)] III; otIs544 [cho-1 fosmid::SL2::mChOpti::h2b] IV	PMID: 26083757
PHX2704	nlp-50(syb2704[nlp-50::t2a::3xnls::gfp]) II	PMID: 34759317
PHX1656	ceh-8(syb1656[ceh-8::GFP)] I	PMID: 32814896
PHX3936	nlp-51(syb3936[nlp-51::SL2::GFP::H2B]) II	PMID: 36178933
OH17148	vab-7(ot959[vab-7::GFP::FLAG]) III	PMID: 32814896
PHX4491	unc-17(syb4491[unc-17::T2A::GFP:H2B]) IV	PMID: 35324425
OP323	unc-119(tm4063) III; wgIs323[ceh-2::TY1::EGFP::3xFLAG unc-119(+)]	PMID: 16990816
SYS633	ujIs113 II; vab-3(dev190([vab-3::mNeonGreen]) X.	PMID: 34312566
OH18089	ceh-43(syb5073[ceh-43::SL2::GFP::H2B] III	PMID: 36178933
OP398	unc-119(tm4063) III; wgIs398[dve-1::TY1::EGFP::3xFLAG + unc-119(+)]	PMID: 16990816
PHX1679	ttx-1(syb1679[ttx-1::GFP]) V	PMID: 32814896
PHX1658	unc-4(syb1658[unc-4::GFP]) II	PMID: 32814896
OP541	unc-119(tm4063) III; wgIs541[cog-1::TY1::EGFP::3xFLAG + unc-119(+)]	PMID: 16990816
OP739	unc-119(tm4063) III; wgIs739[ceh-19::TY1::EGFP::3xFLAG + unc-119(+)]	PMID: 16990816
OP690	unc-119(tm4063) III; wgIs690 [ceh-9::TY1::EGFP::3xFLAG + unc-119(+)]	PMID: 16990816
OH18398	unc-6(syb5064[unc-6::SL2::GFP::H2B] X; otIs669 him-5(e1490)V	This study
OH18111	ttx-1(syb1679 ot1264) V	PMID: 36178933
PHX7566	unc-47(syb7566[unc-47::SL2::GFP:H2B]) III	PMID: 39422452
PHX6486	cat-1(syb6486[cat-1::SL2::gfp::H2B]) X	PMID: 39422452
OH15910	lin-11(ot958[lin-11::GFP::FLAG]) I	PMID: 32814896
OH18821	nlp-18(ot1421[nlp-18::SL2::gfp::h2b]) II	PMID: 39651161
OH18949	lin-11(ot1448) I; nlp-51(syb3936[nlp-51::SL2::GFP::H2B]) II; otIs669 him-5(e1490)V	This study
OH18964	lin-11(ot1454) I; otIs544 [cho-1 fosmid::SL2::mChOpti::h2b]; oig-1 (otIs450 [oig-1 fosmid::SL2::GFP; rol-6(+)])	This study
OH19164	lin-11(ot1495) I; nlp-51(syb3936[nlp-51::SL2::GFP::H2B]) II; ttx-1(syb1679 ot1264) otIs669 him-5(e1490)V	This study
OH19241	lin-11(ot1521) lin-48 (drpIs3 [lin-48p::tdTomato]) I; oig-8(drpIs4 [oig-8p::GFP + pha-1(+)]) II; ttx-1(syb1679 ot1264) him-5(e1490) V	This study
OH19270	ceh-24(syb1608[ceh-24::GFP]) him-5(e1490) V; otIs696	PMID: 32814896
OP68	unc-119(ed3) III; wgIs68[ttx-3::TY1::EGFP::3xFLAG(92C12) + unc-119(+)]) X	PMID: 16990816
PHX2880	ceh-16(syb2709[loxP] syb2880[ceh-16::loxP::GFP]) III	PMID: 36178933
PHX1592	ceh-5(syb1592[ceh-5::GFP]) I	PMID: 32814896
SYS427	ujIs113 II; ceh-10(dev101([mNeonGreen::ceh-10]) III	PMID: 34312566
PHX3242	flp-23(syb3242[flp-23::T2A::3×NLS::GFP])	This study
OH18653	ins-2(syb6543[ins-2:sl2:gfp::h2b]) II; otIs669 V; him-5(e1490) V.	This study
OH17766	ins-3(syb5421[ins-3:SL2:gfp::h2b]) II; otIs669 V; him-5(e1490) V.	PMID: 36178933
OH18772	ins-5(syb6245[ins-5:sl2:gfp::h2b]) II; otIs669 V; him-5(e1490) V.	This study
OH17767	ins-6(syb5463[ins-6:sl2:gfp::h2b]) II; otIs669 V; him-5(e1490) V.	PMID: 36178933
OH10689	otIs355[rab-3p:nls::tagRFP] IV.	PMID: 26291158
OH19210	unc-42(ot1187) V; him-8(e1489) IV.	This study
OH16111	unc-42(ot986[unc-42::GFP]) V	PMID: 34165428
PHX3330	pdf-1(syb3330[pdf-1::t2a::3Xnls::gfp]) III	PMID: 39761329
OH17515	unc-30(ot1186) IV; ric-4(syb2878[ric-4::T2A::3xNLS::GFP]) V	PMID: 35259341
GSH100	unc-30(hzhCR1[unc-30::gfp]) IV)	PMID: 29033363
PHX4413	flp-27(syb4413[flp-27::SL2::GFP::H2B])	PMID: 36178933
NFB1719	mod-5(vlc47[mod-5::T2A::mNeonGreen]) I	PMID: 34232959
OP388	unc-119(ed3) III; wgIs388[lim-6::TY1::EGFP::3xFLAG + unc-119(+)].	PMID: 16990816
PHX3426	ceh-27(syb2714[loxP] syb3286[loxP] syb3426[ceh27::GFP])	PMID: 36178933
SYS473	lim-7(dev125([mNeonGreen::lim-7]) I; ujIs113 II.	PMID: 34312566
OP55	unc-119(ed3) III; wgIs55[mec-3::TY1::EGFP::3xFLAG(9C12) + unc-119(+)]	PMID: 16990816
SD1871	wgIs600 [unc-62::TY1::EGFP::3xFLAG(92C12) + unc-119(+)]	PMID: 16990816
OP748	unc-119(ed3) III; wgIs748 [ceh-6::TY1::EGFP::3xFLAG + unc-119(+)]	PMID: 16990816
OP73	unc-119(ed3) III; wgIs73[ceh-14::TY1::EGFP::3xFLAG + unc-119(+)]	PMID: 16990816
PHX2307	ceh-13(syb2307[ceh-13::mNG::AID]) III	PMID: 38421866
KRA467	lin-39(kas9[lin-39::mNG::AID]) III	PMID: 31902393
PHX6730	syb6730[mab-5::mNG::3xFLAG::AID]	PMID: 38421866
OP54	unc-119(ed3) III; wgIs54[egl-5::TY1::EGFP::3xFLAG + unc-119(+)].	PMID: 16990816
PHX1549	php-3(syb1549[php-3::GFP]) III	This study
PHX2679	nob-1(syb2679[nob-1::GFP]) III.	PMID: 35500030
PHX3240	nlp-1(syb3240[nlp-1::T2A::3×NLS::GFP]) X	This study
PHX4513	flp-5(syb4513 [flp-5::SL2::GFP::H2B]) X	PMID: 35324425
PHX5413	flp-7(syb5413[flp-7:sl2:GFP::H2B]) X	PMID: 39761329
PHX5697	nlp-2(syb5697[nlp-2:sl2:GFP::H2B]) X	PMID: 36178933
PHX5462	ins-18(syb5462[ins-18::SL2::GFP::his-44]) I	https://www.biorxiv.org/content/10.1101/2025.01.06.631508v1
OH17051	ceh-48(ot1125[ceh-48::gfp]) IV	PMID: 35259341
OH16219	ceh-44(ot1015[ceh-44::gfp]) III	PMID: 32814896
OP699	unc-119(tm4063) III; wgIs699 [ceh-43::TY1::EGFP::3xFLAG + unc-119(+)]	PMID: 16990816
OH20074	lim-6(ot1699) X; syb3242[flp-23::T2A::3×NLS::GFP] ;otIs696(NP); him-5(e1490) V	This study
OH20075	lim-6(ot1700) X; unc-47(syb7566[unc-47::SL2::GFP:H2B]) III; otIs696(NP); him-5(e1490) V	This study
OH20076	lim-6(ot1701) X; nlp-18(ot1421[nlp-18::SL2::gfp::h2b]) II; otIs669(NP) him-5(e1490) V	This study
OH20077	lim-6(ot1702) X; nlp-50(syb6148[nlp-50::SL2::gfp::H2B]) II;otIs669(NP) him-5(e1490) V	This study
VT774	unc-36(e251) III; maIs103[mr-1::GFP + unc-36(+)]	PMID: 9716524
OH20172	ttx-1(ot1715); maIs103[mr::GFP + unc-36(+)]	This study
OH20173	ttx-1(ot1716); unc-42(ot986[unc-42::gfp]) V; him-8(e1489) IV	This study
